# SentemQC - A novel and cost-efficient method for quality assurance and quality control of high-resolution frequency sensor data in fresh waters

**DOI:** 10.12688/openreseurope.18134.1

**Published:** 2024-11-07

**Authors:** Sofie Gyritia Madsen van't Veen, Brian Kronvang, Joachim Audet, Thomas Alexander Davidson, Erik Jeppesen, Esben Astrup Kristensen, Søren Erik Larsen, Jane Rosenstand Laugesen, Eti Ester Levi, Anders Nielsen, Peter Mejlhede Andersen

**Affiliations:** 1 Nature and water environment, Envidan A/S, Silkeborg, DK-8600, Denmark; 2 Department of Ecoscience, Aarhus University, Aarhus, DK-8000, Denmark; 3 WaterITech Aps, Skanderborg, DK-8660, Denmark

**Keywords:** SentemQC, quality assurance, quality control, sensor data, high-frequency data, python tool

## Abstract

The growing use of sensors in fresh waters for water quality measurements generates an increasingly large amount of data that requires quality assurance (QA)/quality control (QC) before the results can be exploited. Such a process is often resource-intensive and may not be consistent across users and sensors. SentemQC (QA-QC of high temporal resolution sensor data) is a cost-efficient, and open-source Python approach developed to ensure the quality of sensor data by performing data QA and QC on large volumes of high-frequency (HF) sensor data. The SentemQC method is computationally efficient and features a six-step user-friendly setup for anomaly detection. The method marks anomalies in data using five moving windows. These windows connect each data point to neighboring points, including those further away in the moving window. As a result, the method can mark not only individual outliers but also clusters of anomalies. Our analysis shows that the method is robust for detecting anomalies in HF sensor data from multiple water quality sensors measuring nitrate, turbidity, oxygen, and pH. The sensors were installed in three different freshwater ecosystems (two streams and one lake) and experimental lake mesocosms. Sensor data from the stream stations yielded anomaly percentages of 0.1%, 0.1%, and 0.2%, which were lower than the anomaly percentages of 0.5%, 0.6%, and 0.8% for the sensors in Lake and mesocosms, respectively. While the sensors in this study contained relatively few anomalies (<2%), they may represent a best-case scenario in terms of use and maintenance. SentemQC allows the user to include the individual sensor uncertainty/accuracy when performing QA-QC. However, SentemQC cannot function independently. Additional QA-QC steps are crucial, including calibration of the sensor data to correct for zero offsets and implementation of gap-filling methods prior to the use of the sensor data for determination of final real-time concentrations and load calculations.

## Plain language summary

SentemQC is an open-source Python tool designed to ensure the quality of high-frequency sensor data. The purpose of the method is to clean and perform quality assurance (QA) and quality control (QC) on large volumes of data such as high-frequency data with data points down to every minute. SentemQC is computationally efficient and user-friendly, making it suitable for processing extensive datasets quickly. At the same time, SentemQC features a straightforward five-step setup, making it accessible even for non-experts to perform anomaly detection. The method identifies anomalies using five moving windows calculating the mean or median in each window which is used to detect and mark the anomalies in the dataset. Our results show that the method works well for water quality data (nitrate, turbidity, oxygen, and pH) in various freshwater ecosystems such as lakes, streams, and experimental ponds. This means that the method is robust. However, SentemQC is not standalone as additional steps like calibration and gap-filling in the dataset are necessary for the final cleaning of the data to perform for example load calculations. In summary, SentemQC is a valuable tool for maintaining data quality, however, it should be considered as a part of a broader QA-QC process.

## Introduction

Eutrophication of coastal marine waters and freshwater ecosystems is of global concern as these aquatic systems continue to face significant pressure from human activities and climate change ( European Commission, 2021; Riemann *et al.*, 2016; Schmidt *et al.*, 2023). Therefore, it is crucial to monitor the quality of aquatic ecosystems. Recent advancements in sensor technology have enabled rapid collection of high-resolution sensor data from freshwater systems, offering new opportunities for high-frequency (HF) monitoring ( Bieroza *et al.*, 2023; Rode *et al.*, 2016). Although some sensor technologies may claim to require minimal maintenance and quality assurance (QA)/quality control (QC), integrity and accuracy of HF data are essential and important to avoid producing unreliable and useless data. Therefore, data QA and QC are important, and there is a pressing need to implement standardized QA-QC applications ( Bieroza *et al.*, 2023; Wollheim *et al.*, 2018).

HF sensor monitoring can be useful both regarding scientific understanding of transport pathways of solutes in catchments, hydrochemistry, and aquatic ecology ( Bieroza *et al.*, 2023) and concerning more management aspects through national monitoring programs. In Europe, the chemical status of water bodies (lakes, streams, etc.) is observed with the goal of achieving good status for Europe’s water bodies according to rules in the Water Framework Directive (WFD) ( European Commission, 2000). National monitoring programs typically use traditional infrequent discrete grab sampling techniques, but these may miss crucial events and information ( Cassidy & Jordan, 2011; Levi *et al.*, 2024). To address this issue, HF sensor technology has the potential for future integration into national monitoring programs ( Rode *et al.*, 2016; van Geer *et al.*, 2016) to improve both groundwater and surface water quality management. However, when using HF sensors, there is a need for robust QA and QC, data storage infrastructure, and open data access to enable the investigation of long-term trends. Bieroza *et al.* (2023) advocate for the integration of HF water quality measurements into existing monitoring programs as an important step toward improving water quality, together with increased stakeholder engagement, particularly due to changing environmental conditions and stricter regulatory requirements.

Examples of larger international sensor networks for national monitoring are the U.S. Clean Water Act (CWA), the National Ecological Observatory Network (NEON) in the USA, and the Australian Water Act (AWA) ( Bieroza *et al.*, 2023; Chabbi & Loescher, 2017; Dekker & Hestir, 2012; Edmonds *et al.*, 2022; van Geer *et al.*, 2016). In Europe, several countries use sensors to monitor the quality of their freshwater systems. Ireland has pioneered this approach by incorporating sensors into its Agricultural Catchments Program (ACP) for over a decade ( Jordan *et al.*, 2012). In 2005, the Global Lake Ecological Observatory Network (GLEON) was launched. It is a voluntary international network connecting researchers working with high-resolution sensor data from lakes and reservoirs with the aim to share and communicate their findings ( Read *et al.*, 2016; Weathers *et al.*, 2013).

In most other European countries apart from Ireland, sensors are not fully implemented in national monitoring programs but are often used in special cases, for example in the regional monitoring in Sweden since 2017 ( Fölster *et al.*, 2019) and in a limited number of national river monitoring programs in Norway ( Kaste *et al.*, 2022; Skarbøvik *et al.*, 2023b). The QA-QC of the HF sensor data collected in the different countries varies considerably as shown in a study of turbidity sensor results from 11 different monitoring programs/research projects from six different north-western European countries ( Skarbøvik *et al.*, 2023a). Some countries use manual methods for data QA-QC, but this can be time-consuming and makes it difficult to compare not only the data between the countries but also between the different monitoring programs ( Lannergård *et al.*, 2019; Skarbøvik *et al.*, 2023b; Skarbøvik *et al.*, 2023a; Yang *et al.*, 2023). In addition, large-scale monitoring programs like NEON and GLEON are continuously expanding their geographic reach and sensor equipment ( Schmidt *et al.*, 2023). Furthermore, this enormous amount of HF sensor data requires new smart methods of data quality assurance to ensure that the quality of the collected sensor data is good and acceptable and also follows the FAIR principles ( Wilkinson *et al.*, 2016). In some countries, for example Poland, HF sensor data is QA and QC by private companies applying confidential methods. In these cases, it is difficult to understand how the QA-QC has been performed.

Several methods have been developed for the QA and QC of aquatic sensor data from fresh waters ( Barnett *et al.*, 2012; Belay *et al.*, 2023; Jones *et al.*, 2022; Read *et al.*, 2016; Schmidt *et al.*, 2023; Schmidt & Kerkez, 2023; Talagala *et al.*, 2021). Some methods such as B3 software, ODM Tools Python, the Great Expectations Framework, and the GCE Data Toolbox are mainly based on manual QC and statistics ( Barnett *et al.*, 2012; Gong & Campbell, 2022; Horsburgh *et al.*, 2015; Read *et al.*, 2016; Sheldon, 2008), while others, such as pyhydroqc and SaQC, use more advanced machine learning methods ( Belay *et al.*, 2023; Jones *et al.*, 2022; Leigh *et al.*, 2019; Schmidt *et al.*, 2023; Schmidt & Kerkez, 2023).

Belay *et al.* (2023) performed a numerical assessment of 13 deep-learning algorithms on two time-series of sensor datasets and found that there are still challenges in anomaly detection techniques. This is primarily due to the lack of a universal approach as each method is specifically designed for a particular application ( Belay *et al.*, 2023). Moreover, these machine learning (ML) algorithms can be difficult to follow for the user and are time-consuming to set up and train to fit a specific dataset, and the computational costs involved in training/testing are large ( Belay *et al.*, 2023). Schmidt & Kerkez (2023) also emphasized the need for further research into their QA-QC machine learning method to evaluate the performance of the ML model over longer periods and to assess the generalizability and transferability of ML models trained on various sensor data monitoring identical environmental processes.

Schmidt *et al.* (2023) developed the SaQC method, which includes 32 different CQ functions to flag anomalies. They divided the methods into three overall parts: processing, basic QC tests, and advanced QC tests, where the third part includes machine learning algorithms. This means that the user can choose which QC test they want to use for a specific sensor. For example, they included the Search and TRace AnomalY (STRAY) algorithm for multivariate spike detection. The STRAY algorithm is unsupervised and uses k-nearest neighbor distances for score calculations for anomaly detection ( Talagala *et al.*, 2021). Leigh *et al.* (2019) introduced a ten-step approach for automated detection of anomalies in HF water quality data from in situ sensors. They reviewed and compared different anomaly detection methods such as automated classification rules and several regression and feature-space methods and advocated for a QA-QC approach, that highlighted the importance of understanding end-user requirements and data characteristics to determine the specific type of anomalies and their importance for each sensor type ( Leigh *et al.*, 2019). This framework also allows users to select the most appropriate anomaly detection methods for their specific sensor data.

Anomaly detection concerning HF data has different definitions depending on the focus of the study and input data ( Talagala *et al.*, 2021). Goldstein and Uchida (2016) identify three different types of anomalies: i) Global anomalies, which are easy to detect since they are very different than the rest of the dataset, ii) local anomalies, which can be difficult to detect since they can only be identified as an anomaly when compared to its neighborhood data points, and iii) clusters of anomalies, which can be very difficult to detect as these types of anomalies can look like the normal dataset when comparing each datapoint to its neighborhood data points. The third anomaly type has received less attention compared to the other two categories ( Goldstein & Uchida, 2016; Talagala *et al.*, 2021). In this study, we define an anomaly closely related to the general definition in ( Talagala *et al.*, 2021) that states that an anomaly significantly deviates from the majority of the data, with a much larger distance between typical observations and anomalies compared to the distances among typical observations.

This study aimed to explore the feasibility of implementing a simplified robust QA-QC approach, called SentemQC, to HF sensor data from multiple freshwater systems. The focus was on developing a method that was computationally efficient, transparent, and had a user-friendly setup/interface. The method was designed to identify and mark anomalies and detect errors or patterns in the raw sensor data. SentemQC identifies a data point as an anomaly if it significantly deviates from the other data points within a predefined “moving window”. This deviation is characterized by a much larger distance to the other data points in the moving window, based on statistical calculations such as average, standard deviation, and/or mean. Using SentemQC, all three defined types of anomalies by ( Goldstein & Uchida, 2016) can be detected.

The objectives were i) to develop a simple, open access, and computationally efficient method for QA-QC procedure of HF sensor data called SentemQC; ii) to investigate the robustness of SentemQC by testing it on different sensor measurement types from different aquatic systems and locations; iii) to explore the advantages and disadvantages of the SentemQC program compared to other available programs.

## Study sites

In this study, we examined water quality sensor data from three different freshwater systems ( Table 1).

**Table 1.** Overview of the examples of high-frequency sensor data included in this study. This Table illustrates the data examples in the study used for SentemQC quality assurance and quality control (QA-QC) of high-frequency sensor data.

Type of waterbody	Field site	Data parameters	Measuring period	Measuring frequency
Stream	Lyby-Grønning	Nitrate	2021-05-05 to 2023-01-03	1 min
Stream	Horndrup	Turbidity, nitrate	2021-03-10 to 2023-01-02	1 min
lake	Ormstrup	Dissolved oxygen	2023-07-01 to 2023-10-01	15 min
Lake warming mesocosm experiment (LWME)	Lemming	Dissolved oxygen, pH	2014, 2015, and 2016	30 min

**Figure 1.**
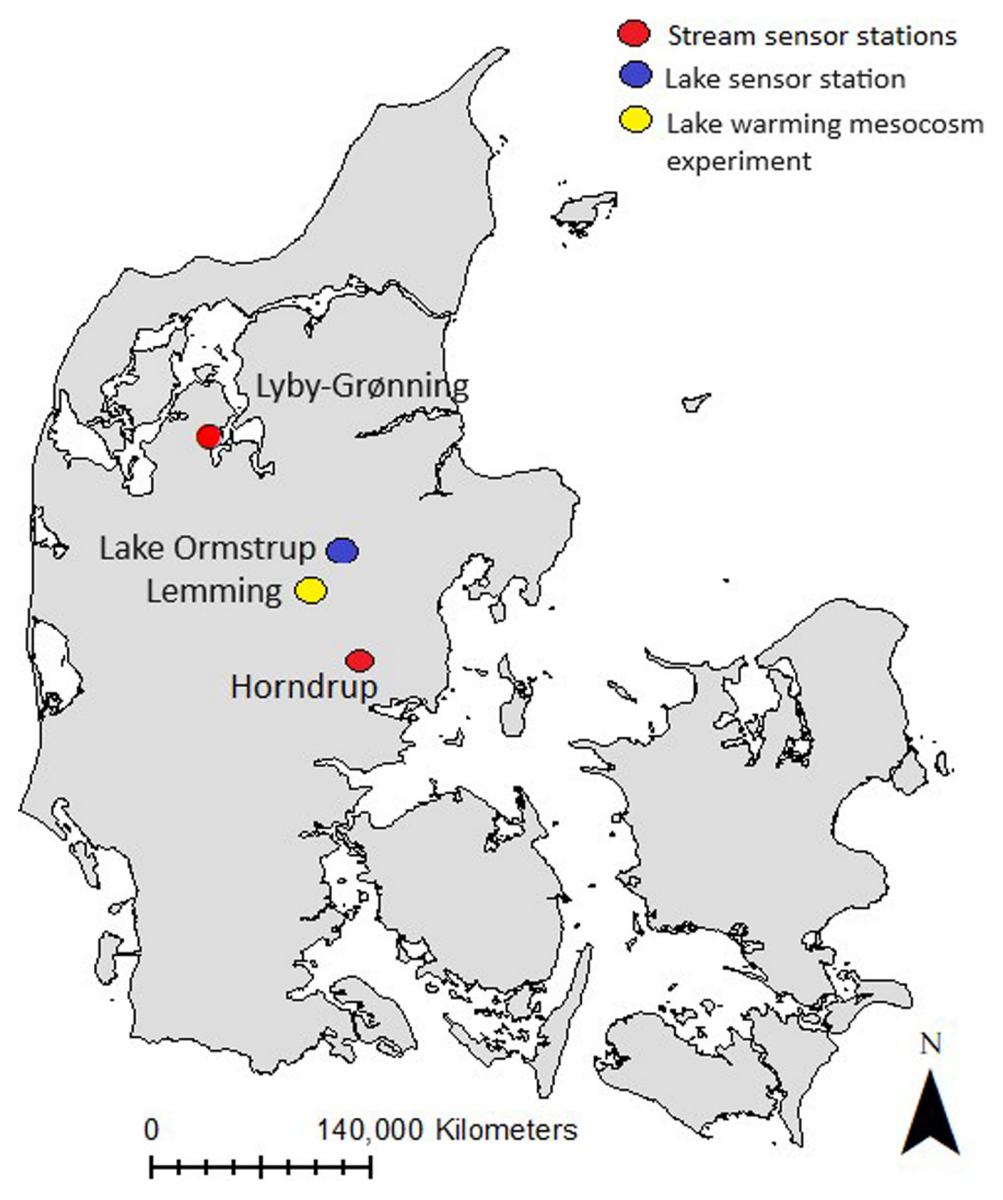
Study sites. The location of the different sensor stations in Denmark. Red dots represent the stream sensor stations Lyby-Grønning Stream and Horndrup Stream, the blue dot represents the lake sensor station Lake Ormstrup, and the yellow dot represents the Lake Warning Mesocosms experiment station in Lemming, Denmark.

The two stream sensor stations were established in Horndrup Stream and Lyby-Grønning Stream in Jutland, Denmark ( Figure 1). The Horndrup stream station covers a catchment area of 5.48 km ^2^, of which 70% is agricultural land and 19% forests ( Levin *et al.*, 2017). The soils are primarily dominated by moraine deposits: fine sand mixed with clay (59%) and fine clay mixed with sand (27%). The stream station is positioned in a part of the stream covered by forest. The Lyby Grønning Stream sensor station covers a catchment area of 11.3 km ^2^ where 84% is agricultural land ( Levin *et al.*, 2017). The soils are dominated by fine sand mixed with clay (47%) and fine clay mixed with sand (52%). Both sensor stations are part of the Danish national monitoring program of the aquatic environment, NOVANA ( Petersen *et al.*, 2021; Windolf *et al.*, 2012).

The lake sensor station is placed in Lake Ormstrup in Jutland, Denmark. The lake is 11 ha and has an average depth of 3.4 m and a hydraulic retention time of >1 year. The lake catchment is small (ca 1 km ^2^), and the lake only has outflow during heavy rainfall events, often in the winter period. The lake is eutrophic with high concentrations of chlorophyll and total phosphorus (TP) ( Davidson *et al.*, 2024; Søndergaard *et al.*, 2023).

The lake warming mesocosm experiment (LWME) sensor stations are located in Jutland, Lemming, Denmark. Here, 24 flow-through outdoor freshwater mesocosms were established in 2003 and are still running in 2024. The overall aim of the mesocosms is to experimentally study how increased temperatures affect shallow lake systems at contrasting nutrient concentrations. A description of the mesocosm setup can be found in Liboriussen *et al.* (2005).

## Data and methods

### Sensor data

This study applied various sensors and data parameters (see Table 1). At the stream stations, nitrate sensors from HACH. In-Situ Nitratax™ plus UV (HACH, Düsseldorf, Germany) was employed, and at Horndrup an additional turbidity sensor from HACH was installed. These sensors gathered data continuously for nearly two years ( Table 1). The sensors were manually cleaned in the field with acid every two weeks and installed with a measuring frequency of 1 minute. The nitrate sensors were *in-situ* Nitratax plus UV purely photometrical sensors that are based on the principle that nitrate and nitrite ions absorb ultraviolet (UV) light at a wavelength of 210 nm. The Nitratax plus UV sensors had the manufacturing series number 1640674 for the nitrate sensor installed in Horndrup Stream, and 2060341 for the nitrate sensor installed in Lyby-Grønning Stream. The sensor sends a light signal every minute. However, there is a built-in automatic function that averages the last three measurements to stabilise the measurements. The sensors also have a built-in wiper technology (double Wiper) for lens cleaning. The double wiper is designed to keep the optical system free of blockages and growth of e.g. algae and deposits ( HACH, 2012; HACH, 2014). The nitrate sensors have a measurement range between 0.1 and 100.0 mg N/L. The precision/uncertainty is 3% or ±0.5 mg N/L ( HACH, 2012; HACH, 2014). Additionally, the nitrate sensor measurements were before using the SentemQC method corrected for zero offsets, which occur linearly for the nitrate sensors. This correction was achieved by using the sensor measurements taken before and after manual cleaning, along with linear interpolation.

The turbidity sensor installed at the Horndrup stream station were *in-situ* optical sensor called Solitax (HACH, Düsseldorf, Germany) with the manufacturing series number 1942974. These sensors determine turbidity by an infrared light measuring technique, which quantifies the intensity of light back-scattered by the water at 90° where the light source is infrared light (860nm). The turbidity was measured in the unit FNU (Formazin Nephelometric Unit), which complies with the European ISO 7027. The turbidity sensor has a measurement range between 0.001 to 4000 FNU and a measuring precision/uncertainty of <5% or ±0.01 FNU ( Table 2) as well as a built-in function stabilizing the measurements. The turbidity sensor has an inferred light source that measures constantly and provides a 1-minute measurement that is an average of the values recorded during the last minute. Additionally, it has a built-in wiper technology (double Wiper) for lens cleaning ( HACH, 2017; HACH, 2018).

**Table 2.** Overview of sensor uncertainty/accuracy for the high-frequency sensor data included in this study. The uncertainty/accuracy of the sensor equipment used to collect the high-frequency sensor data in this study are provided by the manufactories and can be found following the sources in this Table for the different high-frequency sensor data.

Field site	Data parameters	Uncertainty/accuracy	Source
Lyby-Grønning and Horndrup	Nitrate-N	3% of the measured value or ±0.5 mg N/L	( HACH, 2012; HACH, 2014)
Horndrup	Turbidity	Turbidity to 1000 FNU: without calibration <5% of the measured value or ±0.01 FNU	( HACH, 2006)
Ormstrup	Dissolved oxygen	±0.1 mg/L (from 0 to 20 mg/L) ±2% of reading (from 20 to 60 mg/L)	( In-Situ, 2021)
Lemming	Dissolved oxygen	< ±2% of actual value	( OxyGuard International A/S, 1994)
Lemming	pH	0.1 of the actual value is used as a constant. This was chosen because no accuracy is provided in the sensor manual. However, we must be aware that the calibration itself is somewhat uncertain; pH is a log scale and is rounded to 2 decimal places.	( OxyGuard International A/S, 2020)

Dissolved oxygen (DO) and pH sensors was installed in LWME mesocosm named G2 in Lemming. The dissolved oxygen sensor used was an OxyGuard ^®^ two-wire sensor, model 420, and the pH sensor was an OxyGuard light-duty submersion sensor connected to a Manta pH measurement system. Both sensors had a measuring frequency of 30 min, and all sensors were manually calibrated every week. Further description of the sensors and the mesocosm setup can be found in Liboriussen *et al.* (2005), and the uncertainty is provided in Table 2.

The dissolved oxygen sensors (DO) at Lake Ormstrup station 100011 were Aqua TROLL 500 (In-Situ, Fort Collins, CO, USA) multisondes installed near the surface (ca. 1.0 m) and at a deeper-water depth (ca. 3.8 m). The measuring frequency was 15 minutes, and the sensor had a wiper function that automatically cleaned the sensor every 15 minutes before measuring. The uncertainty is ±0.1 mg/L (from 0 to 20 mg/L) and ±2% of reading (from 20 to 60 mg/L) ( Table 2) ( In-Situ, 2021). In addition, the sensors were manually cleaned and calibrated every week. Further description of the sensor setup in Lake Ormstrup can be found in Davidson *et al.* (2024).

### QA-QC method

**Figure 2.**
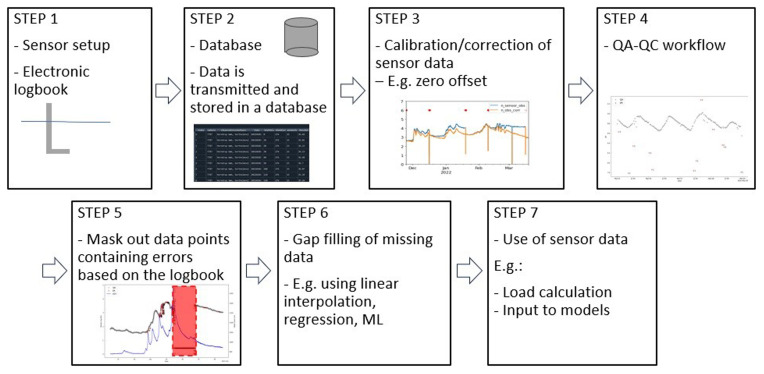
Conceptual model of a hypothetical workflow for data preprocessing high-frequency (HF) sensor data. Step 1 includes an illustration of a sensor installed in a waterbody. Step 2 represents establishing a database containing all HF sensor data. Step 3 represents the calibration of the sensor data with an example showing correction for zero offset of a nitrate sensor. The y-axis is the nitrate-N, and the x-axis is the datetime. The blue line represents the raw data, and the orange line is the corrected sensor data. Step 4 represents and illustrates the quality assurance and quality control (QA-QC) workflow. An example from SentemQC is shown in a Figure where the y-axis is the pH and the x-axis is the datetime. The gray dots are the valid data points and the red dots are the datapoints marked as anomalies. Step 5 represents the use of the electronic logbook to mask out anomalies (red box). Step 6 represents the gab filling of missing data, and step 7 represents the use of the sensor data to for example load calculations.

When working with HF sensor data, we recommend following a specific workflow as outlined in Figure 2. However, QA-QC (quality assurance and quality control) using the method described in this study (Step 4) is just one step of the process and stands out for its robustness and simplicity. The QA-QC method can be applied to various HF sensor data by fine-tuning the parameters as described in the following sections.

### Implementation

The SentemQC method was developed as a freely available Python code to make the method available and easy to apply for future users. The method is free to download from Zenodo at: https://zenodo.org/doi/10.5281/zenodo.13830624 ( vant Veen *et al.*, 2024b) as an open-source package that aims to develop a smart, simple, open-access, and cost-efficient method for the QA-QC procedure of HF sensor data.

The method is based on simple mathematical algorithms using averages, median, and standard deviation calculations. The algorithm uses moving windows of different widths to flag different data patterns as anomalies. To flag large continuous periods of anomalous data, very wide moving windows are applied, yielding a more stable moving average, while individual anomalies and anomalies are flagged with narrow windows. The narrow moving windows can only be used when the large continuous periods of anomalous data are flagged.

The moving windows are all centered, and the calculations for a given data point within the window will therefore have the same number of data points before and after. When a moving window passes through data, calculations are only made on the data points that are not flagged. If a given data point lies outside the window, it will be flagged and excluded in the subsequent window calculations. The width of the individual windows is defined by the user as a given number of data points or a time interval, while the height of the window measurements changes dynamically depending on the data included in the moving window. The upper limit of the moving window for a given data point is the sum of the moving average or median, the product of the moving standard deviation, a predefined factor, and a constant. The lower limit is the difference between the moving average or median, the product of the moving standard deviation, a predefined factor, and a constant. In total, the method uses five windows, and for the fifth moving window, these calculations are weighted with a triangular window. Window three does not calculate moving averages but moving medians to lend some robustness to the method.

The uncertainty of the sensor measurements related to the sensor technology can be defined in the configuration when conducting the parameterization of the QA-QC method as ± in the first two moving windows. The uncertainty can be included as a percentage called “uncertainty_pct” in the parameterization or as a constant called “uncertainty_con”, depending on how the uncertainty is described by the sensor company, see extended data for the final parameterizations ( vant Veen *et al.*, 2024a). For example, for the NITRATAX sensor from HACH, the uncertainty is 3% of the measured value or ±0.5 mg N/L ( HACH, 2012; HACH, 2014). The different uncertainties used in this study are given in extended data 1( vant Veen *et al.*, 2024a).

**Figure 3.**
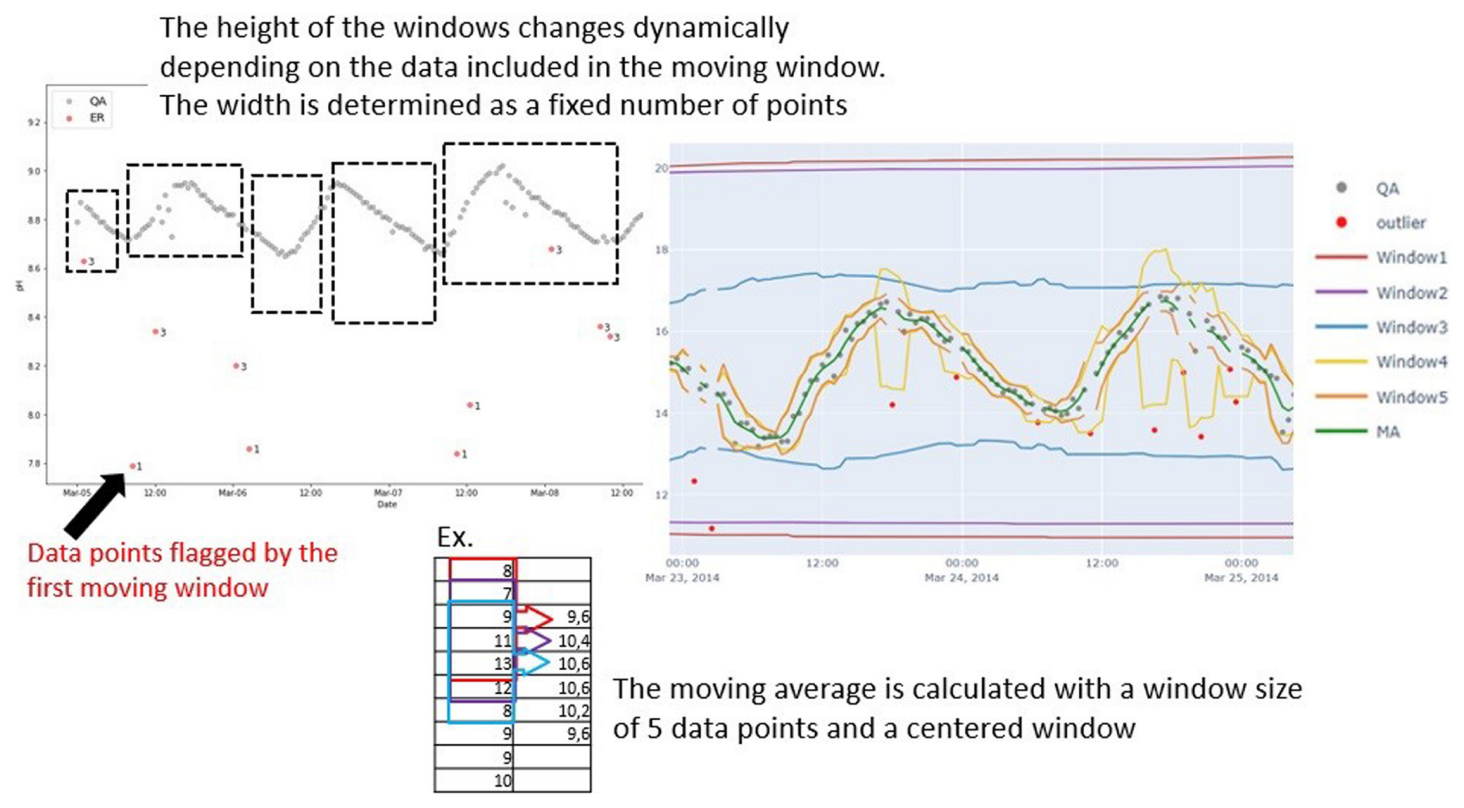
Conceptual model showing the principle behind the SentemQC method. The y-axis is the pH while the x-axis is the datatime. The scatted black boxes represent the different moving windows in the SentemQC program. The gray dots are the valid datapoints, while the red dots are the anomalies flagged by SentemQC. The calculation of the moving average in each window is simplified by the red, blue, and purple boxes.

The method identifies anomalies in the data by comparing the average concentration within a moving window with the established uncertainty range. If a data point falls outside the upper or lower limits of this uncertainty window (represented by ±), it gets flagged as an anomaly. We assume that the uncertainty is found by comparing discrete water grab samples with sensor measurements under controlled conditions in the laboratory and is equally distributed around the measurement point. A conceptual figure illustrating the QA-QC method is seen in Figure 3.

### Operation

SentemQC requires a recent version of Python installed. An environment file that includes the Python versions and packages to run SentemQC is available for download at: https://zenodo.org/doi/10.5281/zenodo.13830624

All files including SentemQC software and the test data for this paper are free to download from Zenodo at: https://zenodo.org/doi/10.5281/zenodo.13830624.

**Figure 4.**
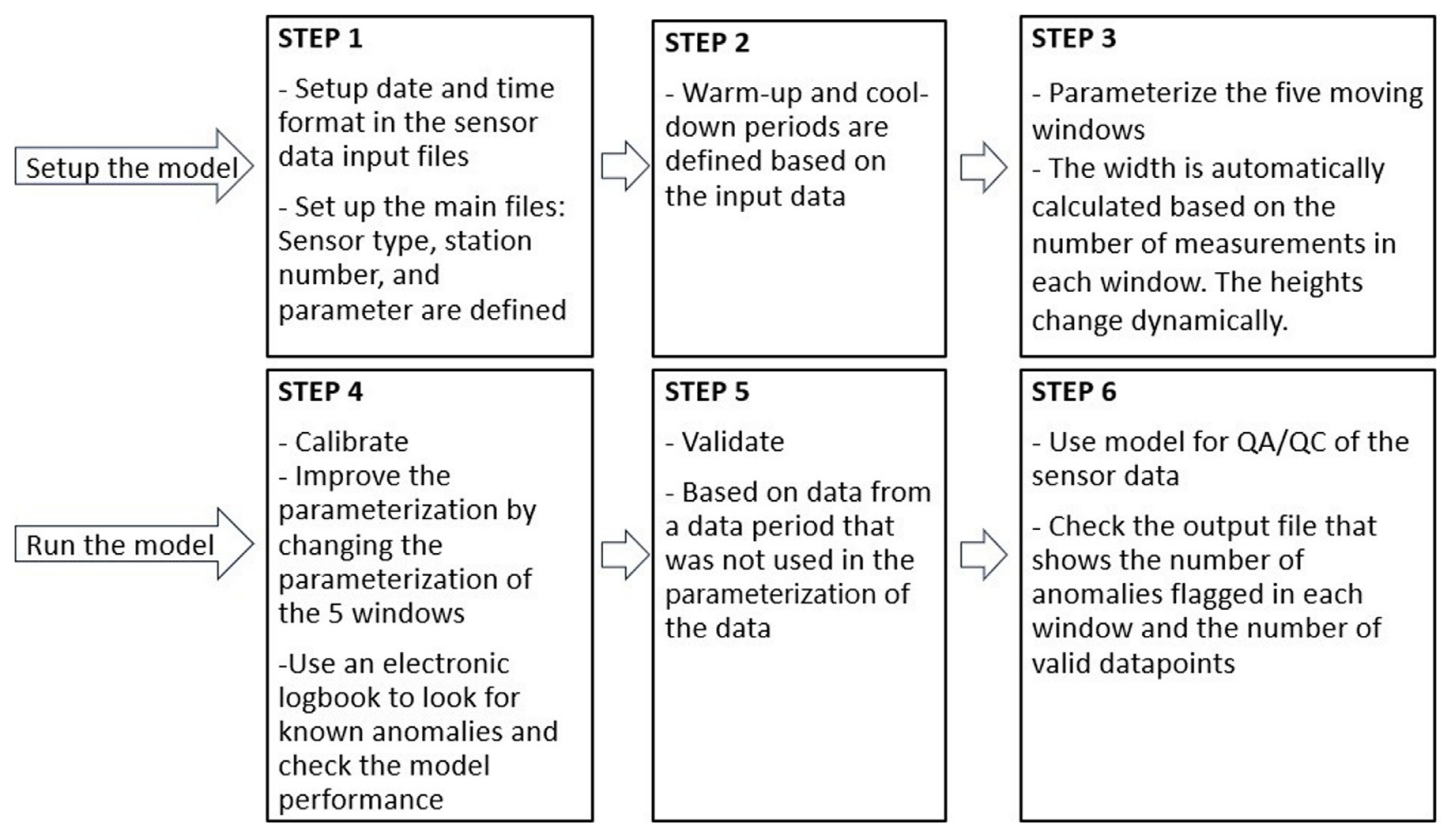
The different steps in the SentemQC quality assurance and quality control (QA-QC) method. The boxes represent the six steps and workflow for data preprocessing in SentemQC.

SentemQC operation flow consists in i) Setting up the SentemQC model, and ii) Running the SentemQC model. In total, SentemQC includes six steps illustrated in Figure 4. SentemQC is set up and controlled by a Python file called Main.py, in which the data period, sensor parameter, and input data are defined. This file loads the QA-QC module and parameterization file. It is important and necessary to have electronic logbooks from the sensor station for the calibration and validation/evaluation of the QA-QC method. In the logbook, each field visit, manual cleaning, calibration, and physical distribution should be noted. Therefore, collaboration between researchers and technicians is important. The QA-QC method produces three outputs: 1) Data Frame. This table contains detailed information about data points identified as anomalies. It includes columns for: The raw data points and whether the data point was flagged as an anomaly or not. If flagged, the specific window that identified it as an anomaly is recorded. Additionally, it shows all valid data points. 2) Time Series Plot. This visual representation displays the entire dataset as a time series. Anomalies are marked out to provide a quick overview of their occurrence. 3) An output file. This file illustrates the total number of flagged anomalies and valid data points in each window. Please download and read the file: SentemQC – Description.pdf for a more detailed description of the operation and folder structure of SentemQC available at https://zenodo.org/doi/10.5281/zenodo.13830624.

### Parameterization of the SentemQC model

The QA-QC method passes through the sensor data five times in total as different moving windows cover different amounts of data. Three of the windows are different, while two are repetitions of the previous window. For example, window 1 has the same parameterization as window 2. The windows are moving and begin directly after each other, which means that the windows do not use each other. An example of the parameterization of the oxygen sensor at the Lemming field site from the library file (*RollWindowStdConfig.json*) and explanations of the components in the parameterization of different windows can be seen in extended data ( vant Veen *et al.*, 2024a).

Parameterization of the QA-QC model’s windows was done individually for the different sensor types installed at the field sites by fine-tuning the model to the specific sensor and field site. For example, in Lyby-Grønning Stream, the nitrate concentration was relatively high compared to the concentration in Horndrup Stream, and some nitrate peaks occurred with a short duration on a daily basis, probably due to point sources. Therefore, it was important that the QA-QC model did not mark out these high peaks as anomalies but still marked out real anomalies. A smaller parameterization/calibration period compared to the entire measuring period was therefore used for the different sensors and field sites to identify the most optimal parameterization for the different windows for the individual sensors.

For Lemming, the year 2014 was chosen as the calibration period because the mesocosms were exposed to some extreme temperature experiments in 2014. Consequently, the parameterization was calibrated to handle extreme conditions.

See the final parameterization and the calibration periods for the individual sensors in extended data 1 ( vant Veen *et al.*, 2024a).

### Results

The results of using the QA-QC method show the robustness and adaptability of various sensor data types. Adaptability is achieved through model parameterization fitted to the specific characteristics of each sensor data set. Several snapshot examples illustrating the sensor data cleaning process enabled by the QA-QC method are presented in the following sections.

### Lake Warming Mesocosm Experiment (LWME) in Lemming

**Figure 5.**
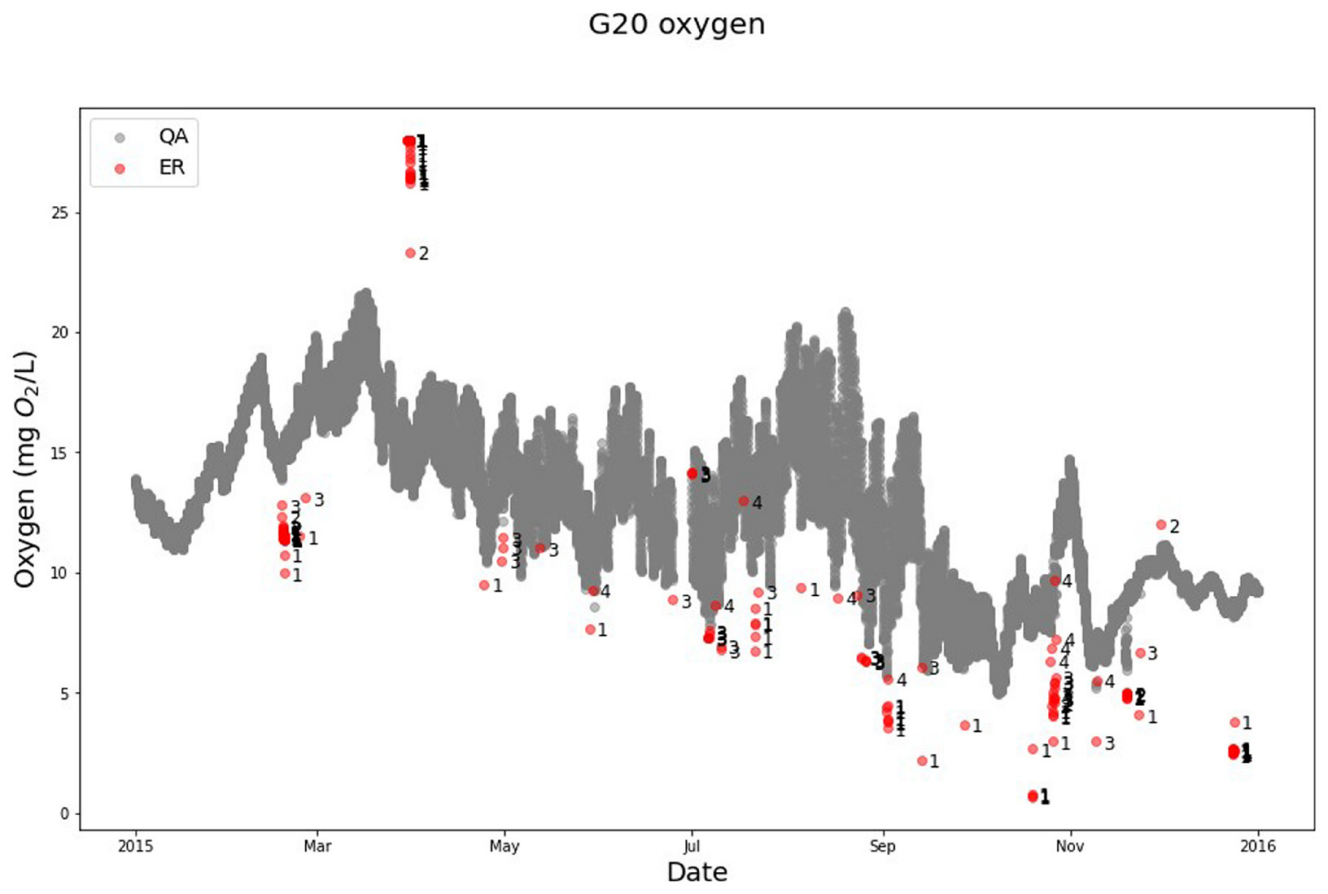
Example of SentemQC of oxygen sensor measurements in the lake warming mesocosm experiment. The example illustrates SentemQC quality assurance and quality control (QA-QC) of Oxygen sensor measurements in the lake warming mesocosm experiment (LWME) in Lemming 2015. The gray dots (QA) are the valid data points. The red dots (ER) are the flagged anomalies by the SentemQC program, and the numbers close to the red dots indicate the moving window flagging the anomaly. “p” means that the data point is a sensor error.

The QA-QC model demonstrates its robustness by its effectiveness in cleaning the oxygen sensor measurements from LWME ( Figure 5). The program identifies and flags various anomalies using different "moving-window rolls” ( Figure 5). It was able to distinguish between different anomaly types like: i) isolated anomalies, which were the most frequent and occurred in most months, ii) sensor error, which was observed in October 2015, indicating a specific issue with the sensor itself, iii) clumps of anomalies, which occurred in March and April 2015, suggesting that external factors potentially affected the measurements, iv) calibration uncertainty, which was found in the April measurement, possibly due to inaccurate calibration of the sensor, and v) unstable sensor anomalies, which appeared around November 2015, indicating sensor malfunction.

Remarkably, the oxygen sensor data revealed more anomalies near the lower limit compared to the upper limit, which is also flagged by the program. This highlights its ability to handle anomalies and distortions, which contributes to its overall robustness. By using the automatic QA-QC program instead of manual cleaning, 10 to 15% more valid measurements were obtained, demonstrating the efficiency and value of the program. Additionally, the calculation time for the QA-QC algorithm for 1.3 million data points was 40 seconds (LWME, Lemming 2015 all mesocosms and parameters).

**Figure 6.**
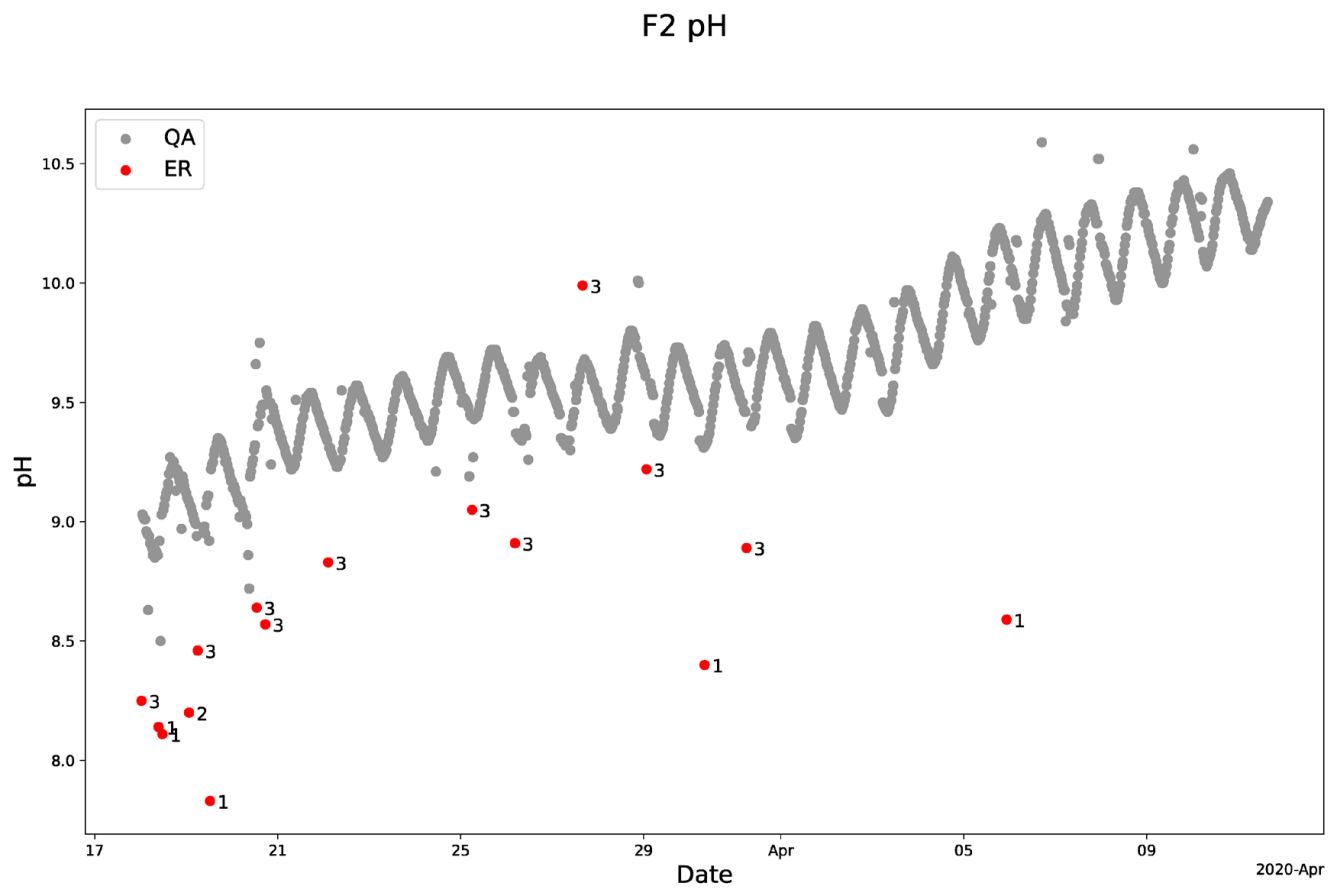
Example of SentemQC of pH sensor measurements in the lake warming mesocosm experiment. The example illustrates SentemQC quality assurance and quality control (QA-QC) of pH sensor measurements in the lake warming mesocosm experiment (LWME) in Lemming 2020. The gray dots (QA) are the valid data points. The red dots (ER) are the flagged anomalies, and the number indicates the moving window flagged the anomaly.

The QA-QC model also demonstrates its robustness by effectively cleaning the pH sensor measurements from Lemming ( Figure 6). Thus, the program identified and flagged various anomalies using different "moving windows” dominated by windows 1 and 3 in the period from March 18 to April 12, 2020 ( Figure 6). As for the oxygen sensor data, the pH sensor data also contained more anomalies near the lower limit compared to the upper limit, which were flagged by the program. This also highlights its ability to handle anomaly distortions, which contributes to the QA-QC programs’ overall robustness ( Figure 6).

However, when zooming in, some individual data points in the data series appeared as potential anomalies although they were not flagged as anomalies by the QA-QC program. This intentional design avoids overly restricting the QA-QC algorithm, which could lead to mistakenly labeling valid data points as anomalies. Therefore, we accepted that anomalies falling inside the upper or lower limits of the uncertainty with values close to the overall trend might not be identified.

### Lyby-Grønning stream and Horndrup stream

**Figure 7.**
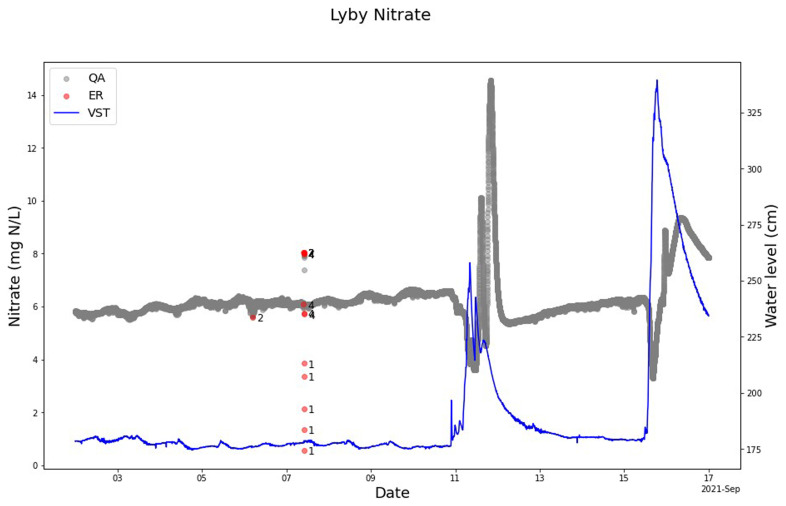
Example of SentemQC of nitrate sensor measurements in Lyby-Grønning Stream. The example illustrates SentemQC quality assurance and quality control (QA-QC) of nitrate sensor measurements in Lyby-Grønning Stream in September 2021. The gray dots (QA) are the valid data points. The red dots (ER) are the flagged anomalies, and the number indicates the moving window flagging the anomaly. The blue line represents the water level in the stream.

The QA-QC model cleaning the nitrate sensor measurement from Lyby Stream is parameterized to include nitrate concentration peaks as valid even if the peak is brief ( Figure 7). We know from discrete samples in the stream that the peaks around September 12, 2021, are valid. This evidences the model’s robustness because it is possible to make adjustments to include such peaks and not mark them as anomalies. At the same time, the QA-QC model could mark the real anomalies that were not real changes in the nitrate concentration measurements in the stream. The high and low concentrations on 7 September 2021 reflect that the sensor was visited and manually cleaned with acid and checked for zero offsets that affect this nitrate sensor before and after manual sensor cleaning ( Figure 7). The SentemQC model perfectly marked out these measurements as anomalies ( Figure 7).

**Figure 8.**
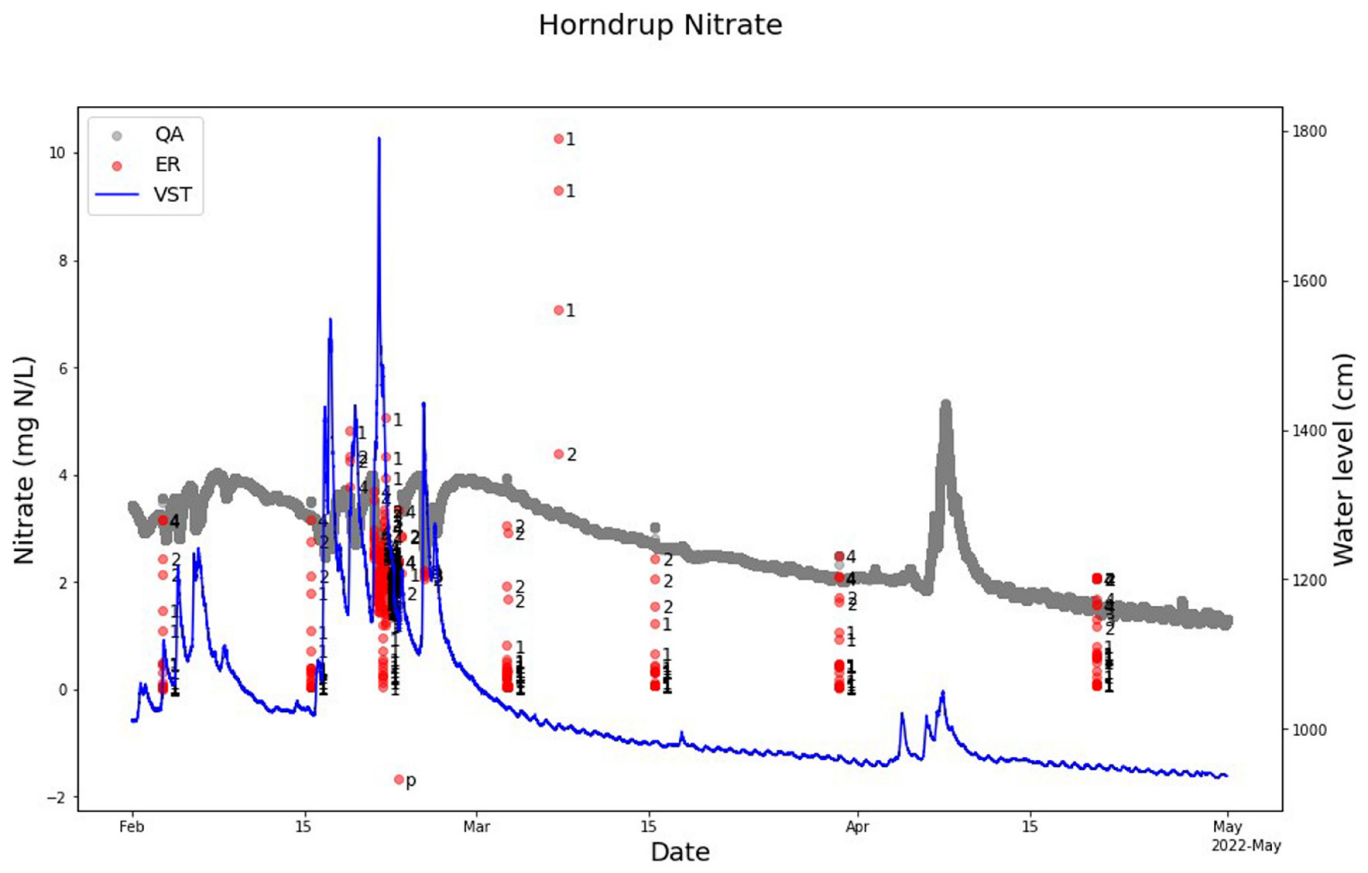
Example of SentemQC of nitrate sensor measurements in Horndrup Stream. The example illustrates SentemQC quality assurance and quality control (QA-QC) of nitrate sensor measurements in Horndrup Stream from February to May 2022. The gray dots (ER) are the valid data points. The red dots (QA) are the flagged anomalies, and the number indicates the moving window that flagged the anomaly. The blue line represents the water level in the stream.

The QA-QC model marked out single anomalies during the manual cleaning of the nitrate sensor measurement from Horndrup Stream on February 15, March 3, 15, and 30, and April 20 ( Figure 8). From February 21 to 23, 2022, the sensor was blocked by branches, leaves, and sand from a heavy rainfall event that transported a lot of sediment to the stream. The QA-QC program marked out these not valid measurements as anomalies. Also, the four single high anomalies around the March 8, 2024, were perfectly marked out as anomalies. In the same period there was no change in the water level ( Figure 8).

**Figure 9.**
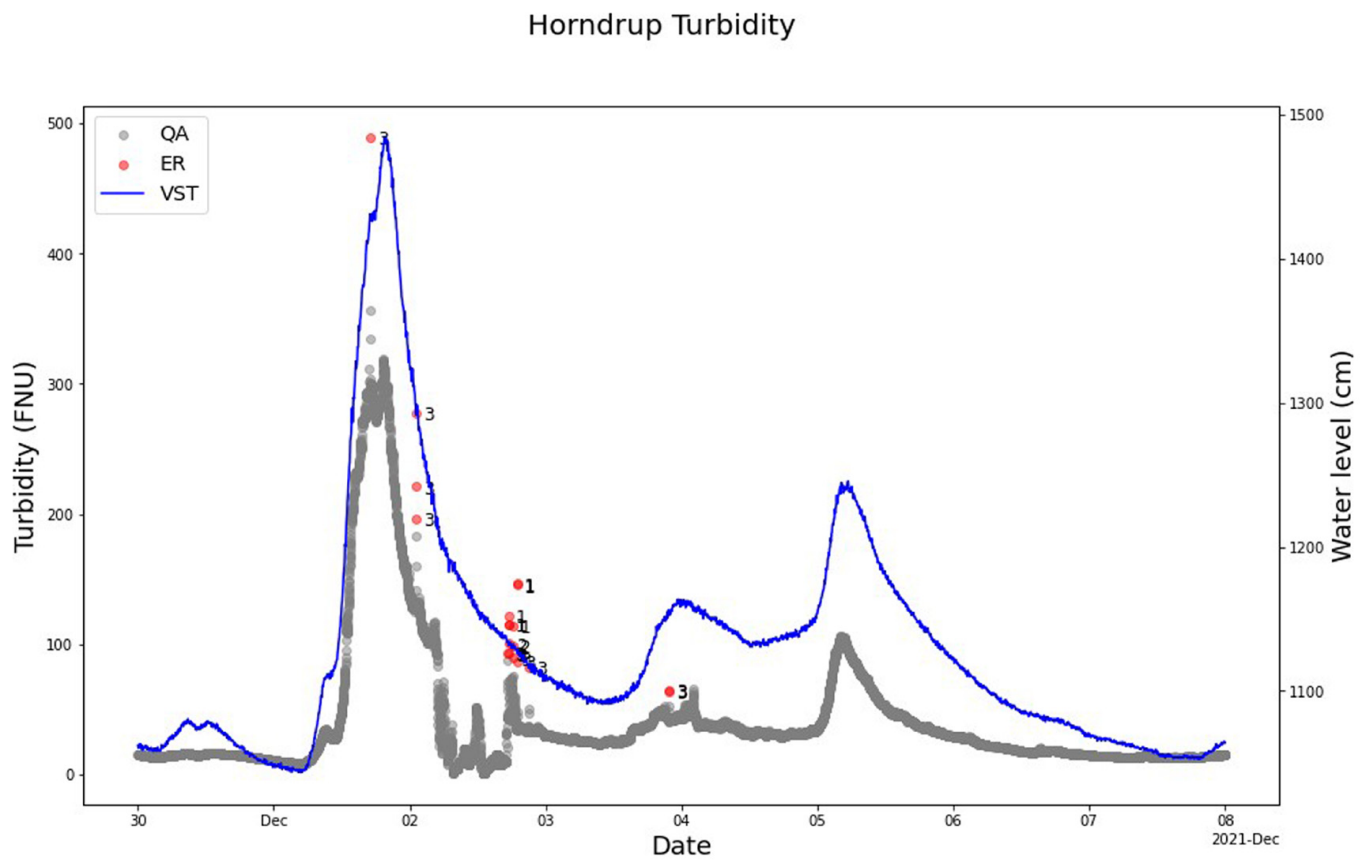
Example of SentemQC of turbidity sensor measurements in Horndrup Stream. The example illustrates SentemQC quality assurance and quality control (QA-QC) of turbidity sensor measurements in Horndrup Stream during a heavy rainfall event at the beginning of December 2021. The red dots (ER) are the flagged anomalies, and the number indicates the moving window that flagged the anomaly. The gray dots (QA) are the valid data points. The blue line represents the water level in the stream.

The use of the QC/QC model cleaning turbidity sensor measurement also showed that the model marked out and captured anomalies and not-valid measuring periods ( Figure 9). The model marked out anomalies such as single noise but also not-valid measurements during a field visit and during manual cleaning of the sensor for the nitrate sensor measurements in Horndrup and Lyby.

**Figure 10.**
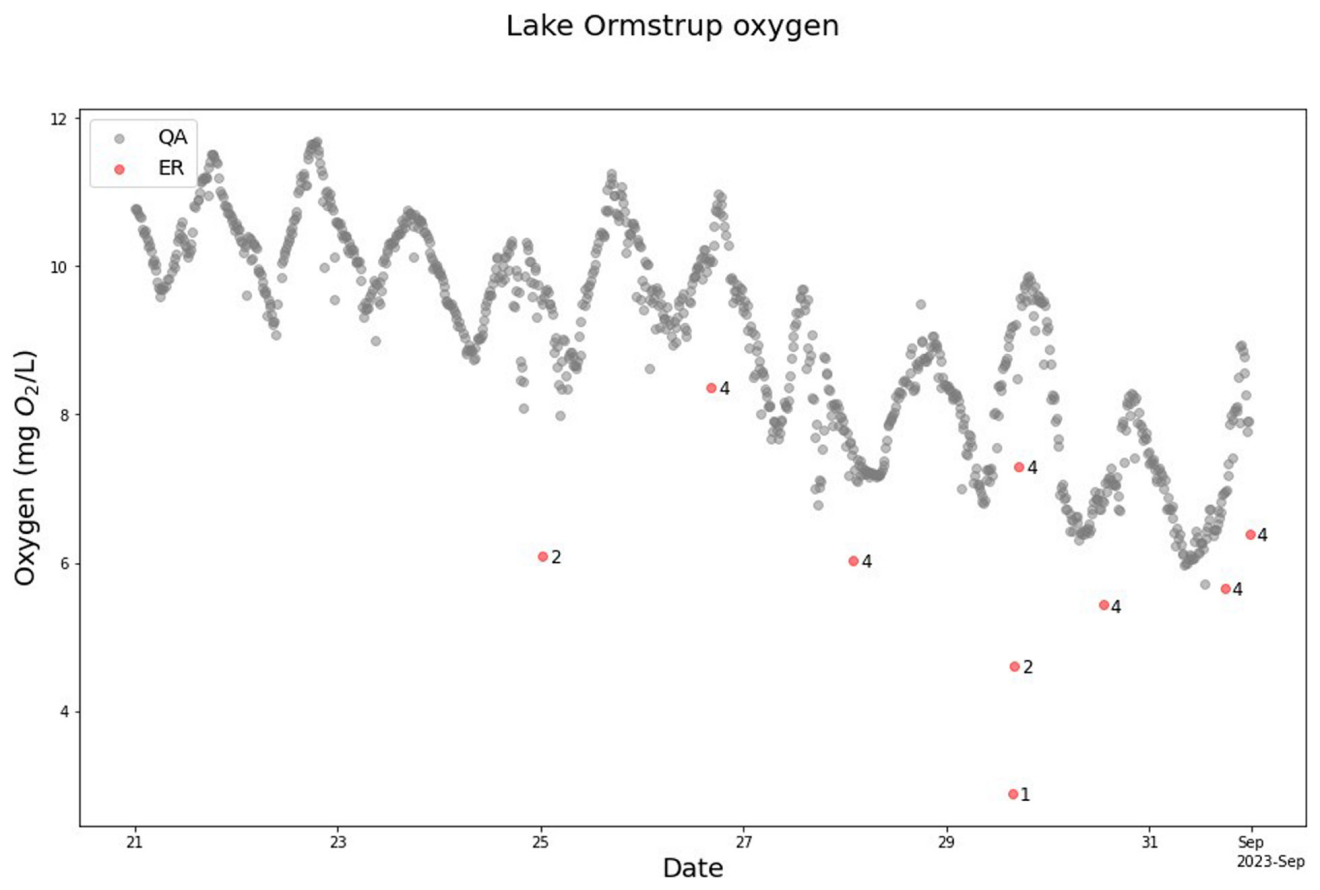
Example of SentemQC of oxygen sensor measurements in Lake Ormstrup. The example illustrate SentemQC quality assurance and quality control (QA-QC) of oxygen sensor measurements from Lake Ormstrup from August 21 to September 1, 2023. The red dots (ER) are the flagged anomalies, and the number indicates the moving window that flagged the anomaly. The gray dots (QA) are the valid data points.

The model parameterization was set to include peaks in the turbidity measurement during, for example, heavy rainfall events where the turbidity is increasing. An example is December 2, 2021, where the model did not mark out the peak but only the single very high concentrations as anomalies ( Figure 9). Window 3 marked out anomalies during this heavy rainfall event, showing that the model can easily be adjusted for cleaning turbidity sensor measurements ( Figure 10).

### Lake Ormstrup

The SentemQC model effectively cleaned the oxygen sensor data from Lake Ormstrup ( Figure 10). It identified anomalies between August 21 and September 1, 2023 ( Figure 10), showing the ability of the model to handle data distortions and improve the data quality. For this oxygen sensor, the SentemQC model also flagged more anomalies near the lower limit than the upper limit ( Figure 10). Some data points might look like anomalies, for example around September 25. However, these are likely caused by wind-induced partial mixing with lower oxygen water, which means that the data points are real.

### Total amount of anomalies in the different sensor data

Table 3 summarizes the number of anomalies flagged by the different moving window settings during QA-QC in SentemQC. The table also shows QA-QC results excluding uncertainty to show the difference and importance of including this parameter ( Table 3). Sensor data from the stream stations yielded a low anomaly rate of 0.1%, 0.1%, and 0.2% including uncertainty, and 0.2%, 0.3%, and 0.5% without uncertainty ( Table 3). This was lower than the anomaly rates observed for sensors in Lake Ormstrup and LWME, which were 0.5%, 0.6%, and 0.8% with uncertainty and 1.6%, 1.4%, and 1.2% without uncertainty, respectively ( Table 3). The differences in anomaly percentages between the different sensor types may be because the sensors installed in the lake and ponds measure oxygen and pH that are considerably more variable parameters than nitrate and turbidity ( Table 3). As expected, SentemQC flagged more anomalies when uncertainty was not included in the program ( Table 3). Additionally, SentemQC also flagged data points as anomalies due to sensor errors that may occur, for example, when sensors are undergoing calibration or maintenance in the laboratory ( vant Veen *et al.*, 2024a). Although SentemQC captured and flagged these points as anomalies, this does not reflect the software's performance in identifying anomalies, and sensor errors were therefore excluded in Table 3.

**Table 3.** Summary of the number of sensor data points flagged as anomalies using SentemQC. This table illustrates a summary of the number of sensor data points flagged as anomalies in the different windows using the quality assurance and quality control (QA-QC) method SentemQC with and without uncertainty included in the method. The number of datapoints flagged and the percentages of anomalies flagged are illustrated.

Sensor/ parameter	Station	Number of data points flagged as anomalies		Percent flagged as anomalies	
		With uncertainty	Without uncertainty	With uncertainty	Without uncertainty
Nitrate-N (mgN/L)	Horndrup Stream	1399	4818	0.2	0.5
Turbidity (FNU)	Horndrup Stream	1298	1944	0.1	0.2
Nitrate-N (mgN/L)	Lyby-Grønning Stream	1025	2663	0.1	0.3
Oxygen (mg/L)	Lake Ormstrup	56	123	0.6	1.4
Oxygen (mg/L)	Lemming LWME	449	643	0.8	1.2
pH	Lemming LWME	84	264	0.5	1.6

There was no general pattern in the number or percentage of flagged anomalies across the different months for any of the sensors investigated, see extended data ( vant Veen *et al.*, 2024a). The nitrate and turbidity sensors almost consistently showed an anomaly rate below 0.4% throughout the year. The sensor measurements therefore seem to be robust and not highly affected by seasonal disruptions during the year. However, the nitrate sensor in Horndrup Stream showed a higher anomaly rate during the winter months (December – February), perhaps due to a heavy bed sediment load, which might have buried the sensor and thus affected its measurements. Throughout the year, sensor height was therefore adjusted in Horndrup Stream based on the amount of rain in the catchment area to ensure that it remained submerged. Unfortunately, in February 2022, the sensor was buried by sediment, and in December 2022 it was positioned too high. In December, the water levels dropped, and the sensor ended up measuring air instead of water, which led to a significant anomaly spike for that specific month ( vant Veen *et al.*, 2024a). Additionally, both the number of anomalies flagged for the pH sensor in Lemming in February and March 2020 at 1.1% and 2.3% and for the oxygen sensor in Lemming in February and March 2015 at 2.9% and 3.3% were relatively high compared to the other months ( vant Veen *et al.*, 2024a).

## Discussion

The main strengths of SentemQC are its simplicity, robustness, and cost-efficiency concerning time resources, low computer power, and the fact that it is freely available for download and use ( vant Veen *et al.*, 2024b). In SentemQC, all data points are connected because the algorithm compares the data points with nearby data points ("neighboring points") using narrow windows and then compares them with points further away using wider moving windows. By examining both nearby and distant data points, this approach ensures that both individual data points (global and local anomalies) and potential clusters of anomalies are effectively flagged. Additionally, SentemQC can provide a QA-QC of 1.3 million sensor data points in 40 seconds. In general, methods such as the rate of change or recursive methods are often used for anomaly detection in dataset ( Barnett *et al.*, 2012; Géron, 2019). However, the rate of change method does not work well for our HF sensor data because the anomalies in the sensor data are sometimes grouped in clusters as described in the Results section. Therefore, this method only identifies and marks out the first and last data point of each cluster as anomalies, thereby missing the remaining anomalies within the group. Using recursive methods was also ineffective for the QA-QC of our sensor data, they were simply too slow and very computer RAM demanding.

The B3 QA software from GLEON ( Barnett *et al.*, 2012; Read *et al.*, 2016) allows the user to investigate the sensor data semi-automatedly and correct the data periods in the QA-QC tools provided by the program ( Barnett *et al.*, 2012; Read *et al.*, 2016). This can be time-consuming as the user has to manually tap and go through the different QA-QC tools and choose which test to use. Additionally, the user must choose between different QA-QC tools and may select a moving average. However, the moving average is only calculated once compared to our five windows, which means that the data points in B3 are only compared to the neighboring data points a single time. This can be inefficient for identifying clusters of anomalies.

Leigh *et al.*'s (2019) ten step-framework also allows users to select the most appropriate anomaly detection methods for their specific sensor data. The first steps involve a collaborative visual examination of the water quality sensor data with end users and identification of the most important anomalies for the specific sensor data. However, this approach can be very resource intensive. In the framework, the user can choose between regression-based and feature-based methods. It requires some level of expertise to choose the right methods, which highlights the importance of clear communication between end-users and anomaly detection developers ( Leigh *et al.*, 2019). Additionally, regression-based methods are semi-supervised and require training, just like ML methods.

The SaQC method allows the user to choose between different QC functions, and there are 32 different QC methods in total. As described in the Introduction, the methods are divided into three overall parts: processing, basic QC tests, and advanced QC tests, where the latter includes ML algorithms ( Schmidt *et al.*, 2023). These methods also require high-level expertise in the specific sensors and the QA-QC methods. Moreover, there may be differences in how the same type of sensor data, such as turbidity, is cleaned. For example, some types can be cleaned using an ML approach, while others can be cleaned using a simpler method based on the user’s individual choice, and this can result in not-harmonized QA-QC approaches. This can render a comparison between the different turbidity data difficult ( Skarbøvik *et al.*, 2023a). Furthermore, the SaQC method’s first part involves preprocessing of the data input as the data needs to be preprocessed before performing the actual QA-QC. This process includes aligning timestamps as well as smoothing and transforming the data ( Schmidt *et al.*, 2023). This is not needed in SentemQC as it can perform QA-QC of all, also shifting time stamps. The input data only needs to contain the correct date-time format. This is an advantage as information can get lost when shifting from 1-minute measuring frequency to 15 minutes.

Additionally, as described in the Introduction, the use of ML approaches for QA and QC often requires substantial computer power and RAM, and it can be difficult to understand the underlying mathematical principles even when spending considerable time on understanding the programming ( Belay *et al.*, 2023). Furthermore, it also takes time to train the ML model compared to the simple SentemQC. Often the responsibility for conducting QA-QC of sensor data quality falls on specialists with in-depth knowledge of the field (domain experts) but less experience with IT technology ( Schmidt *et al.*, 2023). Therefore, the transparency and simplicity of our method are a great strength, making it ideal for specialists even with limited IT knowledge to both use and understand. Furthermore, the user is not required to choose between different methods and can use SentemQC for QA and QC of all types of aquatic sensor data.

Moreover, as pointed out by Belay *et al.* (2023), ML approaches are all very specific for the type of sensor for which they are trained and require input data without noise. This means that it can be difficult to use ML approaches for different types of sensors. Belay *et al.* (2023) consequently came to the conclusion that ML methods are promising but that challenges remain as to the current state-of-the-art multivariate time series anomaly detection techniques. They based their statement on a numerical assessment of 13 deep learning algorithms from two time-series sensor datasets where both their strengths and weaknesses were identified, and the challenges are primarily due to the lack of a universal approach as each method is specifically designed for a particular application ( Belay *et al.*, 2023). 

The studies of Leigh *et al.* (2019) and Schmidt *et al.* (2023) both provide a general workflow for conducting QA-QC of HF sensor data ( Figure 4). Our simple automatic SentemQC could potentially be integrated as an additional QC test within the SaQC program or in Leigh *et al.'s* (2019) framework. As described in the Methods section, the SentemQC program only requires filling in one step to obtain good data quality (step 4, Figure 4). After the QA-QC step (step 5, Figure 4), steps such as implementation of the electronic logbook are needed to mark out periods where the sensor measurement should not be included in the final data time series; for example, if the sensor has been buried in sand or leaves or if the sensor has been taken to the laboratory for control and calibration. SentemQC will automatically mark out the major part of such periods. However, laboratory sensor measurements maybe correct and therefore difficult for the program to capture.

Another step in processing HF sensor data prior to use is gap-filling (step 6, Figure 4), e.g. calibration of the sensor data based on gap samples for correction of zero offsets (step 3, Figure 4), which SentemQC cannot presently perform. For the nitrate sensors in the streams used in this study, a zero-offset of the sensor data needs to be performed based on measurements from before and after manual cleaning of the sensor in the field. These zero offsets are most probably caused by algae, biofilm, and dirt that settle on the sensor lenses when installed in the streams ( van’t Veen *et al.*, 2020), and for these sensors this offset is linear but depends on both stream-specific and season-specific conditions. However, in future work, both the electronic logbook and methods for gap-filling and calibration against gap samples can be implemented in SentemQC.

Schmidt *et al.* (2023) corrected for zero offset of a dissolved organic carbon (DOC) sensor due to biofilm growth and dirt accumulation at the sensor lenses using the SaQC program. The offset exhibited an exponential development, and they used regression to correct for the drift. Therefore, we assume that offsets are site-specific for each sensor and stream.

The use of supporting sensor data parameters could be included in our QA-QC method, e.g. water level, discharge, temperature, or conductivity. In the SaQC program, the function “flagByStray” algorithm is used to calculate k-Nearest Neighbors kNN-scores derived from multiple variables. For example, Schmidt *et al.* (2023) used water level and water temperature as supporting parameters when performing QA of the spectral absorption coefficient at 254 nm (SAC254), which served as a proxy for measuring the dissolved organic carbon (DOC) concentration. However, this required more transformation to normalize the input data. Leigh *et al.* (2019) also tested the use of supporting parameters for QA of environmental data applying feature-based models. For example, kNN-scores determined from multiple variables were used to perform QA of turbidity and conductivity data. They also tested dynamic regression models (RegARIMA models) to forecast the concentration of one parameter based on multiple water quality variables. Yet, both methods can be difficult to follow and require transformation of the input data, and both regression methods and feature-based methods require great computer power for classification ( Leigh *et al.*, 2019).

Another potential parameter to incorporate in SentemQC is sensitivity, for instance for turbidity by comparing it with the water level. During high rainfall events, this should not lead to excessive flagging in the event of a rapid increase in turbidity. Similarly, for nitrate, programming the system to be less sensitive during rainfall events is possible, but comes with a delay, as seen for Horndrup and Lyby-Grønning streams. However, determining the appropriate delay is challenging due to site-specific and seasonal variations, rendering, as described earlier, the method more complicated. Another obvious co-comparison is oxygen and pH, which often follow each other, in lakes and mesocosms. Using supporting parameters would make SentemQC stronger but requires more computational resources. Furthermore, it would make the program less simple and transparent to follow when performing the QA and QC of the sensor data, depending on the type of sensor data and supporting parameters. In addition, Lannergård, Fölster and Futter (2021) found that turbidity sensor measurements were not only correlated with an increase in discharge (Q). Thus, for low-flow events (mean Q < 2 m3/s), the turbidity of the water showed short, repeating increases that were largely independent of changes in the discharge and high turbidity concentrations occurred during periods with decreasing discharge after a high-discharge event. This implies that users should be careful when choosing supporting parameters, for example when performing QA-QC of turbidity sensor data.

As mentioned in the Introduction, there is a need for harmonized and automated QA-QC methods for use on aquatic HF sensor data to allow comparison of data between countries, and because the use of manual methods for QA-QC of large amounts of data is time-consuming ( Bieroza *et al.*, 2023; Skarbøvik *et al.*, 2023a). SentemQC has been tested and used for QA and QC of different sensor data (nitrate, pH, oxygen, turbidity) collected from three different freshwater systems such as streams, lakes, and LWMEs. The method is thus robust, and our study shows that the method can be a sufficient solution for standardizing QA and QC of aquatic sensor data as it is simple, cost-efficient, and open source. 

However, SentemQC could also be used on other types of HF sensor data technologies such as lab-on-a-chip, nanosensors, DNS-based biosensors, or molecular biosensors that all measure with different measuring frequencies and offer different advantages and limitations ( Bieroza *et al.*, 2023) and on sensors applied in wastewater treatment plants where sensor drift is a known problem that may generate high economic costs and energy usage ( Hansen *et al.*, 2022). Former studies have shown that QA-QC of sensor data from wastewater treatment plants can be difficult and sometimes even not possible due to insufficient data and electronic logbooks with missing logs and low data resolution ( Hansen *et al.*, 2022). As pointed out by Scarisbrick-Hauser and Rouse (2007) and Hansen *et al.* (2022), insufficient data quality is a problem in multiple industrial cases. Companies invest in data collection to gain valuable insights, but insufficient data quality often creates a disconnection between intention and reality. We believe that SentemQC with its simplicity and robustness can be of great relevance for gaining more reliable data.

In our study, the use of SentemQC on the sensor data collected at the different sensor stations identified anomalies, flagging from 0.1% to 1.6% of the data as described in the Results section ( Table 3). While the sensors used here contained relatively few anomalies (<2%), they may represent a best-case scenario in term of use and maintenance. Sensors used for monitoring and/industrial application may not be maintained as intensively as under research application and this may increase the potential for anomalies and the need for QA/QC. Without QA and QC, all this data would have been included in subsequent analyses to calculate, for instance, nutrient (N and P) loads. However, by applying SentemQC, we ensured that only reliable and accurate data was considered. For the purpose of comparison, Lannergård, Fölster and Futter (2021) identified anomalies and removed 0.4% of their sensor turbidity measurements in streams, recorded with a frequency of 10 or 15 minutes during six years (2012–2017). They used a manual approach to identify the anomalies by calculating different statistical elements such as standard deviation, mean, maximum, and minimum. The sensor observations were log-transformed, and all turbidity measurements falling outside three standard deviations of the daily mean were identified. However, they also followed several steps during the QC of their sensor data, including calibration using discrete manual samples ( Lannergård *et al.*, 2021).

In SentemQC, we include the uncertainty related to the sensor itself. This uncertainty is given by the manufacturer or company, for example HACH. As described in the Methods section, uncertainty is supposed to be equally distributed around the measurement point. The uncertainty given by the companies is usually stated as plus/minus a percentage to be understood as the relative standard deviation of a large number of analyses where the correct concentration of the chemical substance is known. Normally, analysis results are assumed to have a normal distribution around the true concentration; therefore, it can be discussed if the uncertainty interval should be extended to include plus/minus three standard deviations. If we assume that the sensor measurements exhibit a normal distribution around the true concentration, it covers 96.6% of all outcomes compared to only 68.2% with plus/minus one standard deviation.

**Figure 11.**
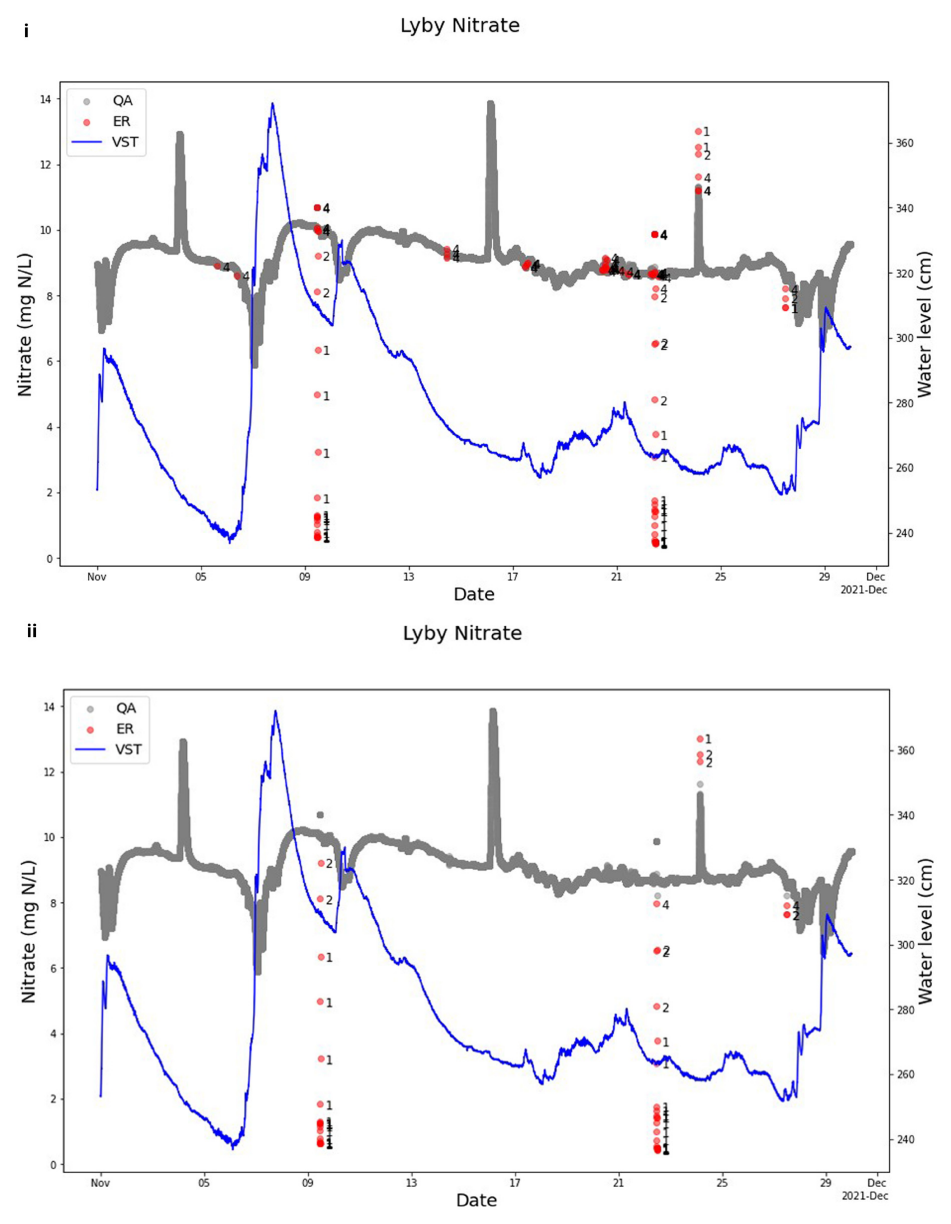
Example of SentemQC with and without uncertainty included from Lyby-Grønning Stream. These two examples illustrate SentemQC quality assurance and quality control (QA-QC) of nitrate sensor measurements from Lyby-Grønning Stream in November 2021 with and without uncertainty included in the method. I) shows the QA-QC of the data without including uncertainty, and Ii) shows the QA-QC of the data including uncertainty. The gray dots (QA) are the valid data points. The red dots (ER) are the flagged anomalies, and the number indicates the moving window that flagged the anomaly. The blue line represents the water level in the stream.

The difference between including and excluding uncertainty when performing SentemQC is given in Table 3, and, as expected, SentemQC flagged more anomalies when excluding the uncertainty ( Table 3). For November 2021, the nitrate measurement in Lyby-Grønning Stream is displayed in Figure 11 as an example. More specifically, the SentemQC flagged 58 anomalies including uncertainty compared to 106 without uncertainty out of 43,200 data points in November 2021 (0.1% vs 0.2%). While the program can identify anomalies, flagging them within the uncertainty range presents challenges as this requires absolute certainty within the uncertain area. However, this is not the case, and the SentemQC program cannot be better than the uncertainty. Therefore, it is important to include the uncertainty. Additionally, none of the currently explored QA-QC methods consider the uncertainty of sensor measurements provided by the sensor companies when performing QA-QC on sensor data.

## Conclusion and perspectives

SentemQC is a cost-efficient and simple program designed to identify and mark out anomalies and detect errors or error patterns in HF sensor data, and this is needed as sensors are neither free of errors nor maintenance-free. SentemQC has, by testing and application, proven effective for QA and QC of sensor data (such as nitrate, pH, oxygen, and turbidity) from various freshwater ecosystems, including streams, lakes, and lake warming mesocosm experiments (LWME). This broad application shows the robustness of SentemQC.

Given its simplicity, cost efficiency, robustness, and open-source availability, SentemQC is a promising solution for standardizing QA and QC of different aquatic sensor data. The SentemQC concept that connects all the data points in the input data by examining both nearby and distant data points ensures that both individual anomalies and potential clusters or groups of anomalies are effectively flagged.

There is a high demand for standardized and automated QA-QC procedures for HF aquatic sensor data to ensure cross-national comparability, and here SentemQC can be useful. Moreover, SentemQC saves workforce compared with the time-intensive manual QA-QC methods, which are impractical for handling enormous amounts of sensor data.

The future development of SentemQC includes integrating the electronic logbook and implementing a calibration procedure for sensor data. Additionally, establishing a mathematical correlation between the dimensions of the moving windows (height and width) for various sensor types together with supporting parameters would improve SentemQC. Furthermore, future developments of SentemQC might include an ML approach and a gap-filling method also using ML. This would enable SentemQC to provide the QA-QC in real time and to do forecasting, which is needed in connection with future warning systems and sensor maintenance. However, this requires considerable effort, complicates the method, and increases the computational demand, as earlier discussed. Currently, we do not have sufficient data to optimize SentemQC by including an ML approach and to retain the universality of the method between different sensor data types.

## Ethics & consent

Ethical approval and consent were not required.

## Data Availability

SentemQC source code, documents, and test datasets examples for this paper are available free of charge from: Zenodo: SentemQC, https://zenodo.org/doi/10.5281/zenodo.13830624 This project contains following data: Python code and test dataset for SentemQC, Data is available under the license Creative Commons Attribution 4.0 International license.

## Extended data

Extended data are available free of charge from

Zenodo: Extended data for the paper: "SentemQC - A novel and cost-efficient method for quality assurance and quality control of high-resolution frequency sensor data in fresh waters", https://doi.org/10.5281/zenodo.13884940

Data is available under the license under Creative Commons Attribution 4.0 International license.

## Software availability

Source code available free of charge from: Zenodo: SentemQC: Python code and test dataset for SentemQC,

URL: SentemQC (zenodo.org) DOI: https://zenodo.org/doi/10.5281/zenodo.13830624

Archived software available from:

URL: SentemQC (zenodo.org) DOI: https://zenodo.org/doi/10.5281/zenodo.13830624

License: SentemQC is under Creative Commons Attribution 4.0 International license.
