# Supplementary material for: SentemQC - A novel and cost-efficient method for quality assurance and quality control of high-resolution frequency sensor data in fresh waters
Source: Open Res Eur. 2025 May 29;4:244. Originally published 2024 Nov 7. [Version 2] doi: 10.12688/openreseurope.18134.2 (PMC11803384; doi:10.12688/openreseurope.18134.2)
Supplement: Peer review history

# Reviewer report: Reviewer response for version 2

**DOI:** 10.21956/openreseurope.22256.r54867

**Published:** 2025-06-30

**Authors:** Arno Hooijboer, Elma Tenner

**Affiliations:** 1 National Institute for Public Health and the Environment, RIVM, Bilthoven, The Netherlands; 2 Environmental quality, National Institute for Public Health and the Environment, Bilthoven, Utrecht, The Netherlands

The authors made significant improvements in the text and incorporated all our comments on the first version. The article is now much clearer on the working and parametrization of  the method.

Is the rationale for developing the new software tool clearly explained?

Yes

Is the description of the software tool technically sound?

Partly

Are sufficient details of the code, methods and analysis (if applicable) provided to allow replication of the software development and its use by others?

Partly

Is sufficient information provided to allow interpretation of the expected output datasets and any results generated using the tool?

Yes

Reviewer Expertise:

Monitoring water quality on farmlands, high frequency water quality monitoring

We confirm that we have read this submission and believe that we have an appropriate level of expertise to confirm that it is of an acceptable scientific standard.

# Reviewer report: Reviewer response for version 2

**DOI:** 10.21956/openreseurope.22256.r54868

**Published:** 2025-06-20

**Authors:** Hongwei Guo

**Affiliations:** 1 Nankai University, Tianjin, China

The authors have carefully revised the manuscript and provided satisfactory responses to my comments raised in the previous round of review. The revisions have substantially improved the quality and clarity of the manuscript. I recommend this manuscript for indexing.

Is the rationale for developing the new software tool clearly explained?

Yes

Is the description of the software tool technically sound?

Yes

Are sufficient details of the code, methods and analysis (if applicable) provided to allow replication of the software development and its use by others?

Partly

Is sufficient information provided to allow interpretation of the expected output datasets and any results generated using the tool?

Yes

Reviewer Expertise:

Water quality of remote sensing

I confirm that I have read this submission and believe that I have an appropriate level of expertise to confirm that it is of an acceptable scientific standard.

# Reviewer report: Reviewer response for version 1

**DOI:** 10.21956/openreseurope.19603.r46097

**Published:** 2024-12-12

**Authors:** Arno Hooijboer, Elma Tenner

**Affiliations:** 1 National Institute for Public Health and the Environment, RIVM, Bilthoven, The Netherlands; 2 Environmental quality, National Institute for Public Health and the Environment, Bilthoven, Utrecht, The Netherlands

The paper describes an automate system in a Python script for QA/QC on high frequent water quality data. The paper is very interesting and easy to read. We endorse the need to do a proper QA and QC to HF data from water quality sensors. It is well-described that for large amount of HF-data quality control should be automated by standardized methods. There are many methods that uses different techniques for QA/QC. It is useful to have an overview of different methods and the authors convince the reader to keep it simple for different reasons.    

 Implementation of the software to the supplied data was easy (within less than one hour). 

 We have some suggestions for improvement:

In general: we got confused about the difference between QA and QC. In our opinion, the data is cleaned afterwards, so it is mainly Quality Control of data (the software is therefore called SentemQC). A definition of both terms and an explanation on which part of the QA and QC the system is useful would make it clear.

In the text

 Page 6 Figure 1: In our print the figure has a low resolution. It would me neater to make it high-res. 

 page 7: “measuring frequency of 30 minutes” should be measuring interval (or frequency of once per 30 minutes). 

 page 8: “the upper limit of the mw for a given data point is the sum” etc. We would appreciate if you provide the formulas, which is clearer than explanation in text. 

 page 9 Figure 4. “Step 4 improve the parametrization by etc”. The power of automated QC is that is performed along standard rules and not randomly. By changing the parametrization doesn't this introduce a human eye which make the QC arbitrary?

 Step 5: The validation is not in the text, shouldn't that be so? what is the size of validation dataset compared to the whole dataset? 

 Page 10: “The QA/QC model passes through the data five times in total”... etc. Since this is the core of the paper and the model we would appreciate it if the author would explain it more in detail. 

 - why is 5 windows chosen and not 4 or 6? 

 - what does each window do? 

 - how do the windows interact with each other? 

 “A smaller parametrization/calibration period” etc: do you mean that the window-sizes are smaller? Please explain how to optimize the parametrization. 

 “the program identifies and flags various anomalies” etc. it would make it more sound if the different examples that are given in the text (i, ii etc) also appear in the figure with arrows or circles. 

 Figure 5: what does QA and stand ER for? In the figure is space enough to explain it more in the legend of the figure  (for example, approved data (grey) and flagged anomalies (red).

 page 12: “the high and low concentrations on 7 September” etc: it is a good sign that the method flags the data during maintenance but in our opinion those data should be disposed form the dataset using the logbook, before doing a QC cleaning of data. 

 In the last sentence before paragraph “Lake Ormstrup”, it refers to Figure 10, which should be Figure 9 in our opinion.

 Discussion: "In SentemQC, all data points are connected because the algorithm compares the data points with nearby data points". Please explain what happens when data gaps appear, is the method also working with missing data?

Downloaded files

 We downloaded the software and the datafiles, however we couldn’t find the YML-file and the VST-level data.

 We recommend to put the Python script file also on GitHub

 In the .pdf with information of the software more explanation should be provided on the parameterization. In the pdf only the parameters are explained of the first window (RollWindowStdConfig.json) we suggest to explain all parameters (and how to define the parameterization). For us it wasn’t clear what settings to use on our own sensor data.

 In the text the authors write that std_factor1 is set to 2.5 but in the example it is 3.

 We look forward to use this approach on our own HF water quality data!

Is the rationale for developing the new software tool clearly explained?

Yes

Is the description of the software tool technically sound?

Partly

Are sufficient details of the code, methods and analysis (if applicable) provided to allow replication of the software development and its use by others?

Partly

Is sufficient information provided to allow interpretation of the expected output datasets and any results generated using the tool?

Yes

Reviewer Expertise:

Monitoring water quality on farmlands, high frequency water quality monitoring

We confirm that we have read this submission and believe that we have an appropriate level of expertise to confirm that it is of an acceptable scientific standard, however we have significant reservations, as outlined above.

# Reviewer report: Reviewer response for version 1

**DOI:** 10.21956/openreseurope.19603.r46103

**Published:** 2024-12-04

**Authors:** Hongwei Guo

**Affiliations:** 1 Nankai University, Tianjin, China

This paper introduces SentemQC, a novel and efficient QA-QC method for water quality high-frequency sensor data, highlighting its simplicity, robustness, and time efficiency. The paper is well-structured and the method is applicable to a variety of water quality scenarios and parameters, showing strong versatility. Specific improvement suggestions:

 1. It is recommended to provide a more detailed algorithm description in the methods section, particularly regarding the rules for multi-window comparison and the detailed logic for anomaly labeling.

 2. Although the article mentions considering sensor measurement uncertainty, there is a lack of detailed discussion on the theoretical basis and practical application of this aspect. It is suggested to add relevant statistical background explanations.

 3. In the results section, it would be helpful to provide a categorical analysis of different types of anomalies (e.g., sensor drift, random noise, clustered anomalies) and their specific manifestations.

 4. SentemQC is described as suitable for various sensor types, but there is insufficient in-depth analysis of the relationship between sensor measurement frequency and data characteristics. It is recommended to supplement this analysis.

 5. The discussion mentions potential future improvements, such as incorporating electronic logs and missing data imputation. It is advised to further specify the technical feasibility of these improvements.

 6. While the simplicity of SentemQC is emphasized, the article lacks a demonstration of the actual operation process. It would be beneficial to include this.

 7. It is suggested to discuss potential scenarios or boundary conditions where SentemQC may not be applicable, such as challenges encountered in complex environments like high biological disturbance waters.

 8. The discussion section suggests combining supporting parameters (e.g., water level, temperature), and it would be valuable to include a case study that validates the improvement in anomaly detection accuracy when incorporating these parameters.

 9. Some references are concentrated, so it is recommended to expand on recent high-quality relevant literature to provide a more comprehensive background to the study.

 10. It is suggested to include a sensitivity analysis of the impact of different threshold settings on the results to validate the robustness of the method.

 11. Quantifying the performance of SentemQC in anomaly detection using metrics such as accuracy, recall, etc., would provide a clearer evaluation of its effectiveness.

 12. Although the discussion section is comprehensive, some parts appear somewhat lengthy. It is recommended to condense and summarize these sections, focusing more on the core arguments.

Is the rationale for developing the new software tool clearly explained?

Yes

Is the description of the software tool technically sound?

Yes

Are sufficient details of the code, methods and analysis (if applicable) provided to allow replication of the software development and its use by others?

Partly

Is sufficient information provided to allow interpretation of the expected output datasets and any results generated using the tool?

Yes

Reviewer Expertise:

Water quality of remote sensing

I confirm that I have read this submission and believe that I have an appropriate level of expertise to confirm that it is of an acceptable scientific standard, however I have significant reservations, as outlined above.

# Response

**Published:** 2025-05-28

**Authors:** Sofie G. M. van't Veen

**Affiliations:** Department of Ecoscience, Aarhus University, Aarhus, Denmark

**Responses to Reviewer Report 2 - Hongwei Guo** **This paper introduces SentemQC, a novel and efficient QA-QC method for water quality high-frequency sensor data, highlighting its simplicity, robustness, and time efficiency.**

**The paper is well-structured and the method is applicable to a variety of water quality scenarios and parameters, showing strong versatility. Specific improvement suggestions:**

*Response: We sincerely thank the reviewer for the thoughtful and encouraging feedback on our manuscript. We are grateful for the time and effort dedicated to reviewing our work and are pleased to hear that the paper was found to be well-structured and that the SentemQC method was well-received. We particularly appreciate the recognition of its simplicity, robustness, and adaptability across various water quality parameters.* *In response to the reviewer’s suggestions, as well as those from the other two review reports, we have revised the manuscript accordingly. The updated version (Version 2) reflects these improvements, and we hope the changes further strengthen the clarity and impact of our work.  The answers specific to review 2 are seen below. The reviewer's original comments are presented in* **bold** *below, followed by our detailed responses in italic. **Thank you for your review, it is much appreciated.***

**1. It is recommended to provide a more detailed algorithm description in the methods section, particularly regarding the rules for multi-window comparison and the detailed logic for anomaly labeling.**

*Response: Thank you for your valuable comment. This is a very good idea. We have revised the 'Implementation' section by including formulas for SentemQC and the moving windows (equations 1 and 2). Additionally, we have expanded the section “Implementation” with a table (Table 3) that provides detailed information about each of the five moving windows. This table includes the calculations, the purpose of the window, and the number of data points included in the window (width).*

**2. Although the article mentions considering sensor measurement uncertainty, there is a lack of detailed discussion on the theoretical basis and practical application of this aspect. It is suggested to add relevant statistical background explanations.**

*Response: Thank you for your valuable comment. We acknowledge the need for a more detailed explanation regarding the sensor measurement uncertainty. In our study, the uncertainty values used in the method are those specified by the manufacturers of the sensors. These are industrial-grade products, and the uncertainty is documented in the respective sensor manuals. While we rely on these manufacturer-provided values, we agree that it could be relevant in future work to investigate whether the stated uncertainty remains valid over time. For instance, after approximately three years of use, wear on the sensor lens may affect measurement accuracy. However, such an investigation falls outside the scope of the present study.*

*Regarding the practical implementation of the sensor uncertainty in the SentemQC method, we have now added a more detailed description of how the uncertainty is incorporated in the five moving windows used in the method. Please refer to the section “Implementation” for this additional explanation.*

**3. In the results section, it would be helpful to provide a categorical analysis of different types of anomalies (e.g., sensor drift, random noise, clustered anomalies) and their specific manifestations.**

*Response: We appreciate the reviewer’s suggestion and agree that a categorical analysis of anomaly types is a valuable addition. While the present version of SentemQC does not explicitly distinguish between different types of anomalies during runtime, we have expanded Figure 5 to illustrate examples of various anomaly types, such as individual anomalies, clustered anomalies, and calibration uncertainty, that the method is capable of flagging. This helps clarify the range of anomalies that SentemQC can detect.*

*We acknowledge that implementing a classification of anomaly types within the method itself would be a meaningful extension. This idea is included in the “Conclusion and perspectives” section in the revised manuscript, where we outline potential directions for future development. However, this functionality is not yet implemented in the present version of SentemQC.*

**4. SentemQC is described as suitable for various sensor types, but there is insufficient in-depth analysis of the relationship between sensor measurement frequency and data characteristics. It is recommended to supplement this analysis.**

*Response: Thank you for your valuable comment. We agree that an in-depth analysis of the relationship between sensor measurement frequency and data characteristics is an interesting and relevant direction. However, we think that such an analysis is beyond the scope of this study. It is worth noting that the datasets used in this study include data from different sensor types with varying measurement frequencies. To perform the suggested analysis thoroughly, we would need to conduct a dedicated iteration for each dataset from each sensor type, which would require a significantly expanded study. We appreciate the suggestion and will consider it for future work, where a more systematic investigation of how measurement frequency influences data characteristics and the performance of SentemQC could be explored.*

**5. The discussion mentions potential future improvements, such as incorporating electronic logs and missing data imputation. It is advised to further specify the technical feasibility of these improvements.**

*Response: Thank you for your comment. We agree that the suggested future improvements, incorporating electronic logs and implementing missing data imputation, are important steps for improving SentemQC. In response to your comment, we have revised the discussion section to include a more detailed consideration of the technical feasibility of incorporating electronic logs and missing data imputation into SentemQC. We now clarify that integrating electronic logs would require a standardized format across users for the electronic logbooks, particularly with respect to datetime formatting, to enable automated masking of known error periods as anomalies. Additionally, we outline several potential approaches to missing data gap-fill, ranging from simple linear interpolation to more advanced methods such as concentration–discharge relationships and machine learning techniques. This highlights the need for further investigation to determine the most suitable implementation strategy. We appreciate your input, which has helped us strengthen the discussion of future improvements.*

**6. While the simplicity of SentemQC is emphasized, the article lacks a demonstration of the actual operation process. It would be beneficial to include this.**

*Response: Thank you for your comment and for pointing this out. We have added supporting information in the 'Implementation' section and the “Parameterization of the SentemQC model” section, which includes supporting information about the different steps in SentenQC and how to optimize the parameterization of the model, the validation of the model, etc. In addition, more information about the operation process and parameterization are also added to the description file ( SentemQC – Description_V12.pdf* *) of SentemQC available together with the program at Zenodo: https://zenodo.org/doi/10.5281/zenodo.13830624* *.*

**7. It is suggested to discuss potential scenarios or boundary conditions where SentemQC may not be applicable, such as challenges encountered in complex environments like high biological disturbance waters.**

*Response: Thank you for your comment. We appreciate the reviewer’s suggestion to discuss potential limitations of SentemQC for more complex environmental datasets. As for now, we have not evaluated datasets where SentemQC failed to perform adequately. We have added a short discussion of the exploration of its limitations to the “Discussion” section. One of the key advantages of SentemQC is that it does not discard any data; instead, it flags data points, allowing for flexible post-processing and interpretation. However, we agree that it would be interesting to investigate its performance on high-frequency datasets with abrupt disturbances, such as those containing bubble-induced signals from gases like methane. These datasets could present unique challenges due to their sudden and irregular nature. That said, we see strong potential for SentemQC in analyzing such datasets that include periods of change or disturbance. In such cases, the method could be particularly valuable when supported by other parameters that could help identify periods with high activity. This could furthermore enhance the robustness of the data cleaning and quality control of the complex environmental sensor datasets.*

**8. The discussion section suggests combining supporting parameters (e.g., water level, temperature), and it would be valuable to include a case study that validates the improvement in anomaly detection accuracy when incorporating these parameters.**

*Thank you for this insightful comment. We agree that incorporating supporting parameters such as water level and temperature has strong potential to enhance the anomaly detection accuracy. In response to your suggestion, we conducted preliminary tests using water level as a supporting parameter. These tests showed good performance. We adjusted the method so that during periods of significant discharge variability, the method dynamically adjusted the standard deviation thresholds within the moving windows. As a result, fewer data points in the dataset were flagged as anomalies in such periods, indicating improved contextual sensitivity. This dynamic adjustment where the upper and lower boundaries of the moving window adapt based on the supporting parameters demonstrates the potential of this approach. However, further testing and validation are needed before full implementation. We are currently developing version 2 of SentemQC, which will include the possibility to include supporting parameters. We expect that this enhancement will significantly improve performance, particularly for datasets like methane, which exhibit rapid fluctuations that make it more challenging to identify anomalies.*

**9. Some references are concentrated, so it is recommended to expand on recent high-quality relevant literature to provide a more comprehensive background to the study.**

*Response: Thank you for the suggestion. In response, we have expanded the literature in the revised manuscript to include several recent high-quality studies. For example, some are focusing on high-frequency sensor monitoring in lakes and other relevant advancements in the field. These additions aim to provide a more comprehensive and up-to-date background for the study.*

**10. It is suggested to include a sensitivity analysis of the impact of different threshold settings on the results to validate the robustness of the method.**

*Response: Thank you for this valuable suggestion. A sensitivity analysis was conducted during the development of SentemQC as a trial-and-error approach. Specifically, we stress-tested the method using the oxygen dataset from Lemming by changing the number of data points included in the five moving windows. For example, the chosen setting of 960 data points in windows 1 and 2 corresponds to a 20-day time series from Lemming, with 48 data points per day. Through this process, we identified a configuration that provided robust performance across different scenarios. When applying SentemQC to other sensor data types from various freshwater environments, we found that the selected window settings gave reliable results. This indicates that the method is not overly sensitive to threshold variations and supports its robustness across the different datasets. We believe that a more in-depth sensitivity analysis of the threshold value (the number of data points in each window defining the width of the window) would add limited value to the study. This is because the “optimal” number of data points is likely to vary depending on the characteristics of each dataset. Our primary objective was to ensure that the SentemQC method is robust and adaptable across a wide range of dataset types. Applying SentemQC, which was originally developed using the oxygen dataset as described above, to other sensor datasets from different freshwater environments demonstrated that the selected parameters delivered reliable quality control, thereby supporting the method’s robustness.*

**11. Quantifying the performance of SentemQC in anomaly detection using metrics such as accuracy, recall, etc., would provide a clearer evaluation of its effectiveness.**

*Response: Thank you for this thoughtful comment. While we agree that performance metrics such as accuracy or recall are commonly used to evaluate anomaly detection methods, they are not directly applicable to SentemQC. This is because SentemQC is not a predictive model in the traditional sense, but rather a rule-based quality control framework designed to flag anomalies. A potential approach to quantitatively assess its performance could involve deploying multiple identical sensors (e.g., five of the same type) in the same aquatic environment (e.g., a lake or stream). By comparing the anomalies flagged across these parallel datasets, one could estimate the consistency and reliability of the SentemQC method. However, we believe that such an experimental setup is beyond the scope of the current study, though we acknowledge it would be a valuable direction for future work.*

**12. Although the discussion section is comprehensive, some parts appear somewhat lengthy. It is recommended to condense and summarize these sections, focusing more on the core arguments.**

*Response: Thank you for this helpful comment. We have carefully reviewed the discussion section and revised several parts to improve clarity and focus in the revised manuscript. Specifically, we have condensed sections that were overly detailed and streamlined the text to better highlight the core arguments and key findings.*

# Reviewer report: Reviewer response for version 1

**DOI:** 10.21956/openreseurope.19603.r46101

**Published:** 2024-11-29

**Authors:** Andrea Lami

**Affiliations:** 1 National Research Council, Water Research Institute (CNR IRSA),, Verbania Pallanza, Italy

This paper deals with an increasing difficulty associated with the technical development of automatic sensor to monitor significative water quality parameters.

 In fact, associated with technological development there is an increasing need to find out a protocol to clean and perform quality assurance (QA) and quality control (QC) on large volumes of data such as high-frequency data with data points down to every minute.

 I found the discussion about the difficulties to filter out outlier well described and appreciate the effort to keep as simple as possible to be user-friendly, making it suitable for processing extensive datasets quickly

 One point that it was not fully clear to me is if you keep track of the number of points that are retained at the end of the filtering process to calculate the mean. Do you consider a lower limit below which mean was not calculated?

 I have some minor comments or suggestions listed here below:

In the Abstract:

 they write “Sensor data from the stream stations yielded anomaly percentages of 0.1%, 0.1%, and 0.2%, which were lower than the anomaly percentages of 0.5%, 0.6%, and 0.8% for the…”

***It is not clear to me to what these percentage are referred. They are the different parameters? You mention 4 parameters and listed 3 percentages.***

In the Introduction:

 a) where they report some examples of HF monitoring in Europe, ***Authors may consider to include the references 1 and 2***

 b) you wrote “32 different CQ functions to flag anomalies” ***It is correct or should be QC***

Study site:

  In Fig. 1 I would add an inset to show the location of Denmark in Europe.

 Is the scale at the bottom of the figure, correct? 140000.00 Km?

In the data and method

***There is any reason why you chose a different frequency in acquisition for DO and pH between LWME and lake Ormstrup?***

***The higher frequency in case of streams respect to lake is because you expect river to be a more dynamic system.***

Results

 In Fig. 3. ***The plot shows pH and Temperature data, but the legend refers only to pH.***

***In the left plot it is stated the moving window have a constant width, but form the plots it seems variable…From the text seems that it is constant within each window and decreasing form W1 to W5? But those shown in the plot do not seems to follow this logic (the first is narrow and the last is wider).***

Is the rationale for developing the new software tool clearly explained?

Yes

Is the description of the software tool technically sound?

Yes

Are sufficient details of the code, methods and analysis (if applicable) provided to allow replication of the software development and its use by others?

Yes

Is sufficient information provided to allow interpretation of the expected output datasets and any results generated using the tool?

Yes

Reviewer Expertise:

limnology

I confirm that I have read this submission and believe that I have an appropriate level of expertise to confirm that it is of an acceptable scientific standard.

## References

[1] : Automated high frequency monitoring of Lake Maggiore through in situ sensors: system design, field test and data quality control. *Journal of Limnology* .2021;80(2) : 10.4081/jlimnol.2021.2011 10.4081/jlimnol.2021.2011

[2] : High-frequency monitoring through in-situ fluorometric sensors: A supporting tool to long-term ecological research on lakes. *Frontiers in Environmental Science* .2023;10: 10.3389/fenvs.2022.1058515 10.3389/fenvs.2022.1058515
